# A Large, Real‐World Cohort Analysis of Arrhythmia Detection and Therapeutic Interventions in Patients With Insertable Cardiac Monitors and Long‐Term Monitoring

**DOI:** 10.1111/jce.70214

**Published:** 2025-12-17

**Authors:** Sandeep A. Saha, Sarah Rosemas, Shantanu Sarkar, Veronica Ramos, Andrew P. Radtke, Shubha Majumder, Mirko De Melis, Jiani Zhou, J. Jason Sims

**Affiliations:** ^1^ Oregon Heart Center Salem Oregon USA; ^2^ Medtronic Cardiac Diagnostics & Services Mounds View Minnesota USA; ^3^ Bakken Research Center Maastricht the Netherlands; ^4^ Grifols Shared Services North America Los Angeles California USA

**Keywords:** arrhythmias, atrial fibrillation, cardiac implantable electronic device, cryptogenic stroke, diagnostic yield, insertable cardiac monitor, syncope

## Abstract

**Background:**

Insertable cardiac monitors (ICMs) provide long‐term continuous monitoring for arrhythmia diagnosis and management for various clinical indications. However, little data exists on comprehensive real‐world arrhythmia diagnostic yield and therapy rates in patients indicated for ICMs with validated artificial intelligence (AI) algorithms enabling large‐scale, automated adjudication of ICM‐detected episodes. We report the largest real‐world analysis of arrhythmia detection as well as medical and procedural therapies in patients with ICMs implanted for guideline‐approved indications with long‐term monitoring.

**Methods:**

Patients who received a Reveal LINQ ICM between October 1, 2016, and June 30, 2020, with ≥ 1 year of follow‐up were identified in two databases (Medtronic CareLink data warehouse, *N* = 12 020, and Optum Clinformatics Data Mart claims database, *N* = 17 037) to analyze arrhythmia detections and therapeutic interventions, respectively. Patients were categorized by clinical indication for ICM placement. All device‐detected ECGs were identified and processed through arrhythmia‐specific AI algorithms. Therapeutic interventions included procedural interventions (cardiovascular implantable electronic device implantation, cardioversions, and ablations) and medication initiation or titration (antiarrhythmics, rate‐control medications, and oral anticoagulants) after ICM implant.

**Results:**

Mean (SD) follow‐up in the CareLink and Clinformatics claims databases was 24.6 (12.7) and 40.8 (15.6) months, respectively. Of the 12 020 patients in the arrhythmia detection analysis, 7284 (60.6%) had ≥ 1 arrhythmia detected (56.3% in the suspected AF population; 80.1% in the AF management population), and 376 (28.9%) had ≥ 2 arrhythmias detected during long‐term follow‐up. Among syncope patients with arrhythmia(s) detected, 71.2% had a finding other than pause/bradycardia; 50.4% of cryptogenic stroke patients and 62.6% of AF management patients with arrhythmias had ≥ 1 finding other than AF. Of the 17 037 patients in the therapeutic interventions analysis, 9820 (57.6%) had a therapeutic action post‐ICM insertion, with 25% of all patients receiving a procedural intervention, and > 50% undergoing a medication adjustment. Mean (SD) follow‐up to first arrhythmia detection was 7 (9) months. Mean (SD) duration from ICM insertion to therapeutic action was 13 (13) months for procedures and 7 (11) months for medication initiation.

**Conclusions:**

Long‐term continuous monitoring with ICMs enables identification of multiple arrhythmias that may have otherwise remained undetected and rules out arrhythmias in ~40% regardless of indication. Medication adjustments and/or procedural interventions related to the management of arrhythmias were observed in over half of ICM recipients during long‐term follow‐up.

## Graphical abstract

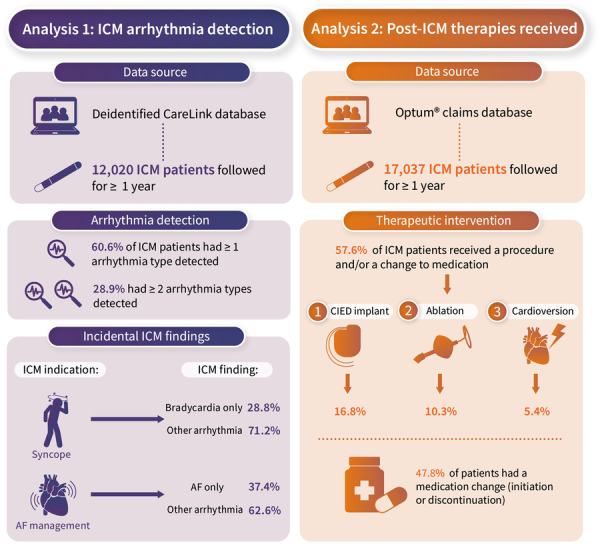

**Abbreviations**
AF: atrial fibrillation
CIEDs: cardiovascular implantable electronic devices
ICM: insertable cardiac monitor

## 1 Introduction

Over the past 20 years, insertable cardiac monitors (ICMs) have been utilized for clinical conditions where long‐term, continuous cardiac arrhythmia monitoring is needed to obtain a definitive diagnosis and to make subsequent therapy decisions. ICMs have been shown to have a higher diagnostic yield and lead to a greater rate of arrhythmia therapies compared to short‐term, noninvasive arrhythmia monitoring in a variety of clinical situations [1, 2, 3, 4, 5, 6, 7, 8, 9, 10]. As such, current clinical guidelines support the use of ICMs for a number of indications, including unexplained syncope, cryptogenic stroke, palpitations, and ventricular arrhythmias [11, 12]. With greater awareness of the clinical utility of ICMs and the ease of implanting these devices in the office or ambulatory surgical centers [13], the use of ICMs has dramatically increased [14]. In addition, with studies such as STROKE‐AF potentially supporting expansion of the clinical indications for ICMs in stroke patients, and resurging interest in monitoring patients undergoing ablation for atrial fibrillation (AF) [15, 16], the clinical use of ICMs will continue to increase. The increasing volume of ICM implants, coupled with their improved diagnostic capabilities, has also led to a deluge of monitoring data generated from these devices, and in‐office device clinics and arrhythmia monitoring centers have to sift through enormous amounts of data and provide timely, actionable information to clinicians to enable them to make therapeutic decisions [4, 17, 18]. To facilitate this, diagnostic algorithms in modern ICMs now incorporate artificial intelligence (AI)‐based arrhythmia adjudication to improve diagnostic accuracy and have been shown to significantly reduce “false alerts.” [19, 20, 21, 22, 23]

ICMs also have the ability to detect incidental arrhythmias in real‐world clinical practice, which were either unexpected or thought to be clinically unrelated based on the clinical indication for implantation. The clinical implications of both anticipated and incidentally detected arrhythmias across various patient populations have not been comprehensively studied. In our study, we explored these questions using a novel approach. Utilizing a large‐scale, robust arrhythmia monitoring database with AI‐adjudicated ECG data and a linked administrative claims database, we sought to characterize the diagnostic yield for all AI‐adjudicated arrhythmias detected in patients implanted with ICMs across multiple guideline‐approved clinical indications. We then conducted a separate analysis of arrhythmia‐related medical and procedural therapeutic interventions that occurred following ICM implantation in these patient populations.

## 2 Materials and Methods

### 2.1 Data Source

This retrospective cohort study utilized data from two large, nationally represented deidentified databases: Medtronic CareLink data warehouse and Optum Clinformatics Data Mart (CDM) claims database (Central Illustration 1, 1). Optum's CDM encompasses administrative health claims data from a vast population, including members of large commercial and Medicare Advantage health plans, covering approximately 19 million individuals annually across all 50 states in the United States. The CareLink data warehouse encompasses both device registration and continuous heart rhythm monitoring data for all patients with Medtronic cardiovascular implantable electronic devices (CIEDs) since 2007.

**Central_Illustration 1:**
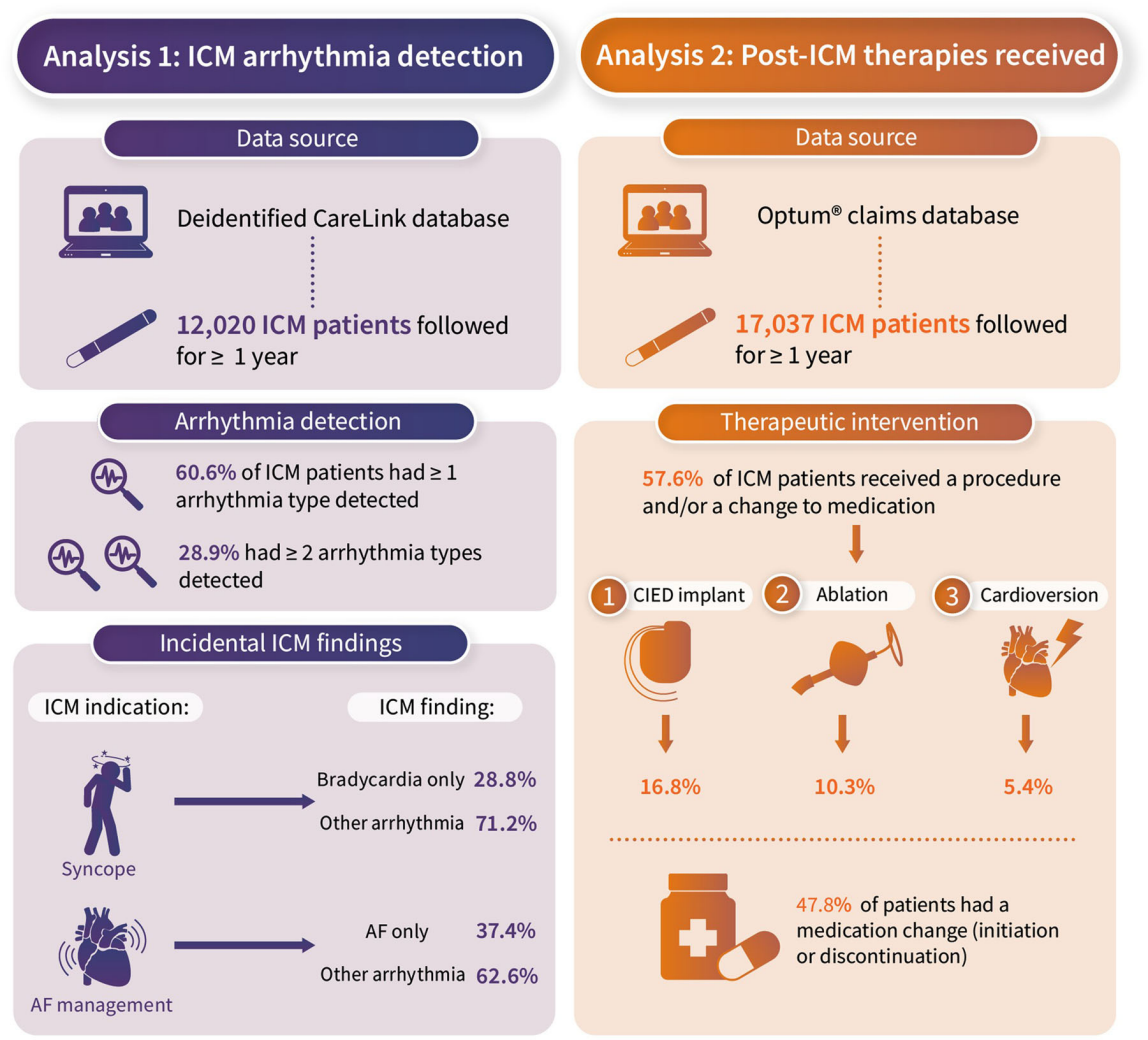
ICM arrhythmia detection and therapeutic interventions. AF, atrial fibrillation; CIEDs, cardiovascular implantable electronic devices; ICM, insertable cardiac monitor.

The CDM claims data warehouse is deterministically linked to the CareLink device registry via seven encrypted personal identifiers. The data set was assessed, relative to the Health Insurance Portability and Accountability Act of 1996 (HIPAA), by a third‐party compliance partner and deemed low risk of identification via expert determination. The final deidentified data set included device registration and CDM data that was joined together by a non‐identifiable patient identifier.

### 2.2 Study Cohorts

Patients who received a Reveal LINQ ICM between October 1, 2016, and June 30, 2020, were identified in each of the two databases (CareLink data warehouse and Optum CDM) to analyze arrhythmia detections and therapeutic interventions, respectively. Henceforth, these analyses will be referred to as the (1) arrhythmia detection analysis and (2) therapeutic intervention analysis.

The cohort for the CareLink‐based arrhythmia detection analysis is a subset of the Optum CDM‐based therapeutic intervention analysis, as both cohorts are linked to the CareLink device registry (and thus both are sampled from LINQ ICM implants during the same time period). However, as the CDM claims data did not have comprehensive linkage to the CareLink heart rhythm monitoring data, any CareLink monitoring follow‐up inclusion criteria were not applied to these patients.

### 2.3 Study Design

**Figure 1:**
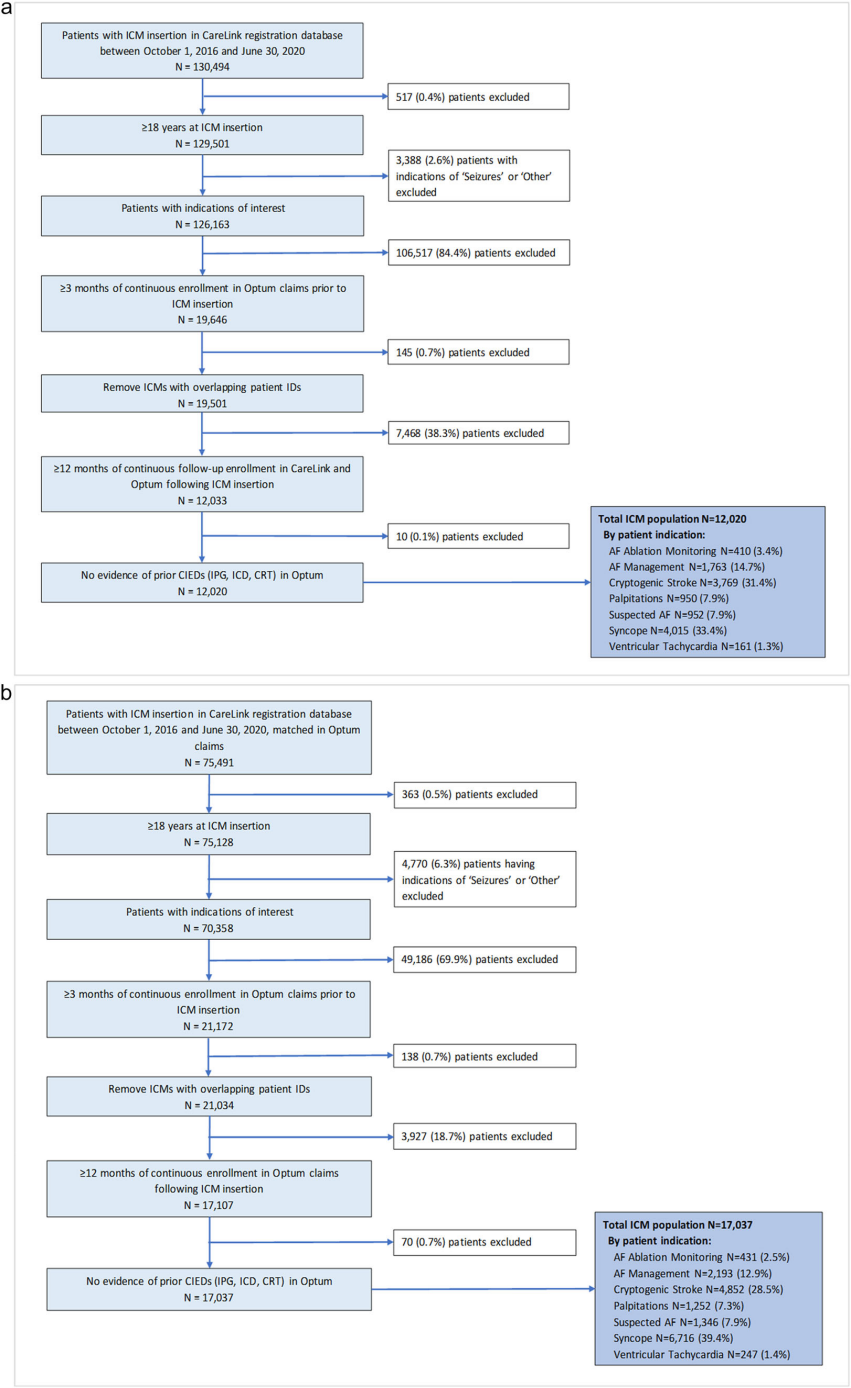
(a) Patient selection flowchart for arrhythmia detection analysis using Carelink Data. (b) Patient selection flowchart for therapeutic interventions analysis using Optum CDM Claims Data. CIEDs, cardiovascular implantable electronic devices; CRT, cardiac resynchronization therapy; ICD, implantable cardioverter defibrillator; ICM, insertable cardiac monitor; IPG, implantable pulse generator; Optum CDM, Optum Clinformatics Data Mart claims database.

The date of ICM insertion was categorized as the index date. The 6 months pre‐ICM insertion were classified as the baseline period to capture patient characteristics. To be included in the study, patients were required to have at least 3 months of continuous enrollment before ICM insertion and continuous enrollment for at least 12 months post‐ICM insertion date. Patients were excluded if they were < 18 years old; had a prior CIED; the clinical indication for monitoring in the device registration database was listed as “other,” “seizures,” or was missing; or had < 12 months of follow‐up post‐ICM insertion (Figure 1, 1).

Patients were characterized based on the indication for ICM as listed in the device registration at implant, and were organized into two patient subgroups:

2.4

- Suspected arrhythmias: Patients with cryptogenic stroke, palpitations, suspected AF, unexplained syncope, and ventricular tachycardia (VT).

2.5

- Management of known AF: Patients with known AF who received an ICM for AF management or postablation AF monitoring.

Patients were followed through the end of continuous insurance eligibility, death, or the end of data availability (December 31, 2021), whichever came first.

### 2.6 Study Outcomes

#### 2.6.1 Analysis #1: Arrhythmia Detection Analysis

This study assessed detection rates for the various types of arrhythmias detected by ICM, including bradycardia, pause, AF, atrial tachycardia (AT), supraventricular tachycardia (SVT), monomorphic ventricular tachycardia (MVT), polymorphic ventricular tachycardia (PVT), and ventricular fibrillation (VF). ECG rhythm data for all device‐detected arrhythmia alerts during the follow‐up period were retrieved from CareLink. All tachycardia and pause arrhythmias were processed through previously validated arrhythmia‐specific AI algorithms, which were trained using data from over 60 000 and 24 000 adjudicated ECG episodes, respectively, and validated with an independent set of 5025 and 2766 adjudicated ECGs to reduce false positive arrhythmia detections by 88.2% and 97.4% while preserving > 99% of true arrhythmia detections [19, 20, 21, 22]. The positive predictive value (PPV) of the four AI algorithms used to adjudicate arrhythmia was in the 90%–95% range. The adjudication of bradycardia episodes relied on high‐accuracy existing device logic. The nominal duration at which each of the arrhythmias can be detected by ICM (and which are also required by the AI in the adjudication of true episodes) is as follows: pause ≥ 3 s; bradycardia = 4 consecutive beats slower than 2 s resulting in a minimum overall duration of 8 s; AF ≥ 2 min, VT = 16 consecutive RR intervals < VT detection interval (which is 220 beats/min minus the age of patient) resulting in a minimum duration of 4 s; FVT = 30 of 40 recent intervals < 260 ms resulting in a minimum duration of 8 s. The first true arrhythmia findings of a given arrhythmia type were captured and assessed, and the time from ICM insertion to true arrhythmia detection was characterized across all arrhythmia types.

#### 2.6.2 Analysis #2: Therapeutic Interventions Analysis

Arrhythmia‐related therapeutic interventions were characterized post‐ICM insertion during follow‐up, utilizing ICD‐10‐PCS and CPT procedure codes (Table S1). The ICM insertion date was considered as index date to characterize time from ICM insertion to therapeutic intervention. Interventions included the following:

- Procedures: including CIED implants, cardioversions, and cardiac ablations for AF, SVT, or VT.
- Medications: Oral anticoagulants (OACs), antiarrhythmic drugs (AADs), or rate control medications (including beta‐blockers and calcium channel blockers). Medication changes were defined as follows:
  ‐ *Medication initiation*: a patient had a new claim for medication fill after their ICM insertion, but no claims for fills during the 6‐month pre‐ICM baseline period.
  ‐ *Medication discontinuation*: a patient had evidence of at least one medication claim during the baseline period, but no evidence of medication claims within the patient's last 100 days of post‐ICM follow‐up.
  ‐ *Medication change:* either a discontinuation or initiation occurred.

### 2.7 Statistical Analysis

Patient demographics and comorbidities during the baseline period were summarized separately for patients based on the clinical indication for monitoring. Continuous variables were presented as mean ± standard deviation (SD) or median + interquartile range (IQR), and categorical variables were expressed as counts and percentages.

For the overall ICM population and stratified by clinical indication for monitoring, the proportion of patients with arrhythmia findings, categorized by the type and number of distinct arrhythmia types detected per patient, was calculated. The duration from ICM insertion to the first ICM‐detected arrhythmia type, second ICM‐detected arrhythmia type, and last ICM‐detected arrhythmia type were calculated and presented as mean (SD), median (IQR), and range.

The proportion of patients with each type of arrhythmia‐related therapeutic intervention (including procedures and medication changes) post‐ICM implant was determined, and the time from ICM insertion to therapeutic intervention was calculated and presented as mean (SD), median (IQR), and range.

## 3 Results

### 3.1 Patient Baseline Characteristics

#### 3.1.1 Analysis #1: Arrhythmia Detection Analysis

This analysis encompassed a total of 12 020 patients (average age 70.2 ± 12.2 years; 52.4% female) who received an ICM between October 1, 2016, and June 30, 2020 (Figure 1, 1). The most prevalent baseline comorbidities included hypertension (74.1%), ischemic stroke (37.2%), coronary artery disease (28.9%), and diabetes mellitus (27.3%) (Table 1). Among the 9847 (81.9%) patients monitored for diagnosis of suspected arrhythmias, 4015 (40.8%) had unexplained syncope, 3769 (38.3%) had cryptogenic stroke, 950 (9.7%) had palpitations, 952 (9.7%) were monitored for suspected AF, and 161 (1.6%) for VT. Among the 2173 (18.1%) patients monitored for management of known AF, 1763 (81.1%) were indicated for AF management, and 410 (18.9%) for post‐AF ablation monitoring.

**Table 1:** Baseline characteristics in arrhythmia detection and therapeutic intervention analyses.

ICM Indication	Overall ICM population	Known AF indications		Suspected arrhythmia indications				
		AF ablation monitoring	AF Mgmt	CS	Palpitations	Susp AF	Unexplained syncope	Ventricular tachycardia
Analysis								
Arrhythmia detection analysis	12 020	410	1763	3769	950	952	4015	161
Therapeutic intervention analysis	17 037	431	2193	4852	1252	1346	6716	247
Demographics								
Mean age, years	70.2 (12.2)	67.2 (10.3)	70.3 (10.0)	71.0 (10.7)	64.0 (14.9)	70.9 (10.3)	71.1 (13.4)	64.6 (14.9)
	70.8 (11.9)	69.4 (9.6)	71 (9.7)	71.4 (10.4)	64.9 (14.4)	71.4 (10.1)	71.6 (12.8)	64.5 (16.3)
Female	6296 (52.4%)	149 (36.3%)	792 (44.9%)	1964 (52.1%)	626 (65.9%)	484 (50.8%)	2206 (54.9%)	75 (46.6%)
	8924 (52.4%)	178 (41.3%)	973 (44.4%)	2554 (52.6%)	814 (65.0%)	682 (50.7%)	3611 (53.8%)	112 (45.3%)
Comorbidities (3 months before ICM implant)								
Cardiomyopathy	812 (6.8%)	69 (16.8%)	139 (7.9%)	188 (5.0%)	42 (4.4%)	64 (6.7%)	278 (6.9%)	32 (19.9%)
	1234 (7.2%)	62 (14.4%)	199 (9.1%)	266 (5.5%)	73 (5.8%)	96 (7.1%)	493 (7.3%)	45 (18.2%)
Congestive HF	1396 (11.6%)	68 (16.6%)	249 (14.1%)	387 (10.3%)	71 (7.5%)	89 (9.4%)	513 (12.8%)	19 (11.8%)
	2212 (13.0%)	83 (19.3%)	360 (16.4%)	544 (11.2%)	113 (9.0%)	175 (13.0%)	906 (13.5%)	31 (12.6%)
CAD	3447 (28.9%)	118 (28.8%)	486 (27.6%)	1000 (26.5%)	221 (23.3%)	275 (28.9%)	1286 (32.0%)	61 (37.9%)
	5074 (29.8%)	131 (30.4%)	636 (29.0%)	1293 (26.7%)	308 (24.6%)	402 (29.9%)	2219 (33%)	85 (34.4%)
Diabetes	3286 (27.3%)	93 (22.7%)	428 (24.3%)	1249 (33.1%)	187 (19.7%)	231 (24.3%)	1059 (26.4%)	39 (24.2%)
	4744 (27.9%)	86 (20.0%)	561 (25.6%)	1617 (33.3%)	256 (20.5%)	336 (25.0%)	1834 (27.3%)	54 (21.9%)
Hypertension	8903 (74.1%)	277 (67.6%)	1227 (69.6%)	3072 (81.5%)	580 (61.1%)	679 (71.3%)	2956 (73.6%)	112 (69.6%)
	12 585 (73.9%)	298 (69.1%)	1537 (70.1%)	3900 (80.4%)	766 (61.2%)	987 (73.3%)	4928 (73.4%)	169 (68.4%)
Ischemic stroke	4468 (37.2%)	34 (8.3%)	185 (10.5%)	3240 (86.0%)	85 (9.0%)	230 (24.2%)	677 (16.9%)	17 (10.6%)
	5923 (34.8%)	35 (8.1%)	247 (11.3%)	4053 (83.5%)	114 (9.1%)	319 (23.7%)	1130 (16.8%)	25 (10.1%)
Mitral valve disease	126 (1.1%)	4 (1.0%)	24 (1.4%)	45 (1.2%)	5 (0.5%)	8 (0.8%)	39 (1.0%)	1 (0.6%)
	189 (1.1%)	6 (1.4%)	26 (1.2%)	65 (1.3%)	9 (0.7%)	13 (1.0%)	66 (1.0%)	4 (1.6%)
PVD	1436 (12.0%)	28 (6.8%)	178 (10.1%)	534 (14.2%)	80 (8.4%)	97 (10.2%)	493 (12.3%)	26 (16.2%)
	2093 (12.3%)	31 (7.2%)	232 (10.6%)	702 (14.5%)	115 (9.2%)	151 (11.2%)	831 (12.4%)	31 (12.6%)
Pulmonary disease	1627 (13.5%)	37 (9.0%)	198 (11.2%)	486 (12.9%)	107 (11.3%)	125 (13.1%)	654 (16.3%)	20 (12.4%)
	2453 (14.4%)	45 (10.4%)	283 (12.9%)	662 (13.6%)	139 (11.1%)	197 (14.6%)	1090 (16.2%)	37 (15.0%)
Sleep apnea	2183 (18.2%)	113 (27.6%)	416 (23.6%)	603 (16.0%)	182 (19.2%)	200 (21.0%)	637 (15.9%)	32 (19.9%)
	2920 (17.1%)	107 (24.8%)	500 (22.8%)	739 (15.2%)	222 (17.7%)	279 (20.7%)	1029 (15.3%)	44 (17.8%)
Thyroid disease	2409 (20.0%)	65 (15.9%)	317 (18.0%)	784 (20.8%)	175 (18.4%)	181 (19.0%)	867 (21.6%)	20 (12.4%)
	3415 (20.0%)	76 (17.6%)	403 (18.4%)	970 (20.0%)	233 (18.6%)	262 (19.5%)	1437 (21.4%)	34 (13.8%)
CCI, mean(SD)	1.3 (1.7)	1.0 (1.5)	1.2 (1.6)	1.5 (1.7)	0.9 (1.5)	1.2 (1.6)	1.4 (1.7)	1.3 (1.7)
	1.4 (1.7)	1.1 (1.5)	1.3 (1.7)	1.5 (1.7)	1 (1.5)	1.3 (1.7)	1.4 (1.7)	1.2 (1.7)
CHA2DS2‐VASc score, mean(SD)	3.7 (1.9)	2.6 (1.6)	3.0 (1.6)	4.9 (1.6)	2.6 (1.6)	3.3 (1.7)	3.4 (1.7)	2.7 (1.8)
	3.66 (1.87)	2.75 (1.54)	3.01 (1.67)	4.79 (1.71)	2.66 (1.59)	3.39 (1.73)	3.38 (1.74)	2.68 (1.77)
Follow‐up time								
Months, mean(SD)	24.56 (12.7)	29.1 (11.3)	26.8 (11.8)	23.7 (12.5)	27.2 (12.1)	25.8 (12.2)	23.3 (13.1)	26.6 (11.5)
	40.8 (15.6)	42 (25.6)	39.6 (14.4)	38.4 (15.6)	42 (16.8)	42 (15.6)	40.8(16.8)	39.6 (15.6)

Abbreviations: AF, atrial fibrillation; CAD, coronary artery disease; CCI, Charlson comorbidity index; CS, cryptogenic stroke; HF, heart failure; Mgmt, management; PVD, peripheral vascular disease; Susp, suspected; VT, ventricular tachycardia.

The overall mean (SD) follow‐up duration was 24.6 (12.7) months, with a median (IQR) duration of 24.5 (15.2–35.5) months. The mean follow‐up duration for each indication ranged from 23.3 (13.1) to 29.1 (11.3) months (Table 1). Patients were predominantly Medicare Advantage patients across all indications (Table S2).

#### 3.1.2 Analysis #2: Therapeutic Interventions Analysis

The therapeutic interventions analysis included 17 037 patients (average age 70.8 ± 11.9 years; 52.4% female) who received an ICM between October 1, 2016, and June 30, 2020 (Figure 1, 1). The most prevalent baseline comorbidities included hypertension (73.9%), ischemic stroke (34.8%), coronary artery disease (29.8%), and diabetes (27.9%). Among the 14 413 (85%) patients who were indicated for the diagnosis of suspected arrhythmias, 6716 (39.4%) had unexplained syncope, 4852 (28.5%) had cryptogenic stroke, 1252 had palpitations (7.35%), 1346 (7.9%) had suspected AF, and 247 (1.4%) had VT. Among the 2624 patients indicated for management of known AF, 2193 (12.9%) were indicated for AF management and 431 (2.5%) for postablation AF monitoring.

The overall mean (SD) follow‐up duration was 40.8 (15.6) months, with a median duration of 39.3 (30.0–51.6) months. The mean follow‐up duration for each indication varied from 38.4 (15.6) to 42.0 (25.6) months (Table 1).

### 3.2 Outcomes During Follow‐Up

#### 3.2.1 Analysis #1: Arrhythmia Detection Analysis

##### 3.2.1.1 All ICM Patients

**Figure 2:**
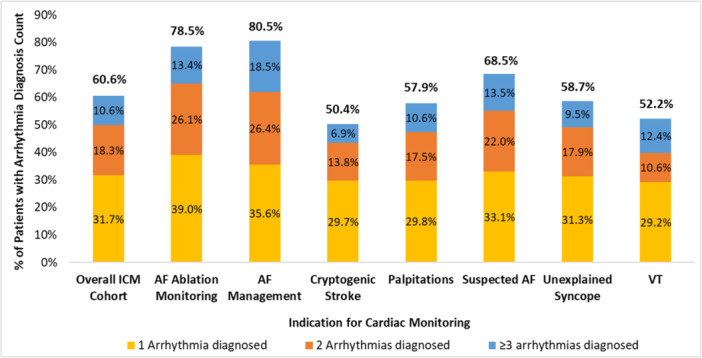
Total number of distinct arrhythmias detected by ICM per patient, by patient indication for monitoring. AF, atrial fibrillation; ICM, insertable cardiac monitor; VT, ventricular tachycardia.

**Figure 3:**
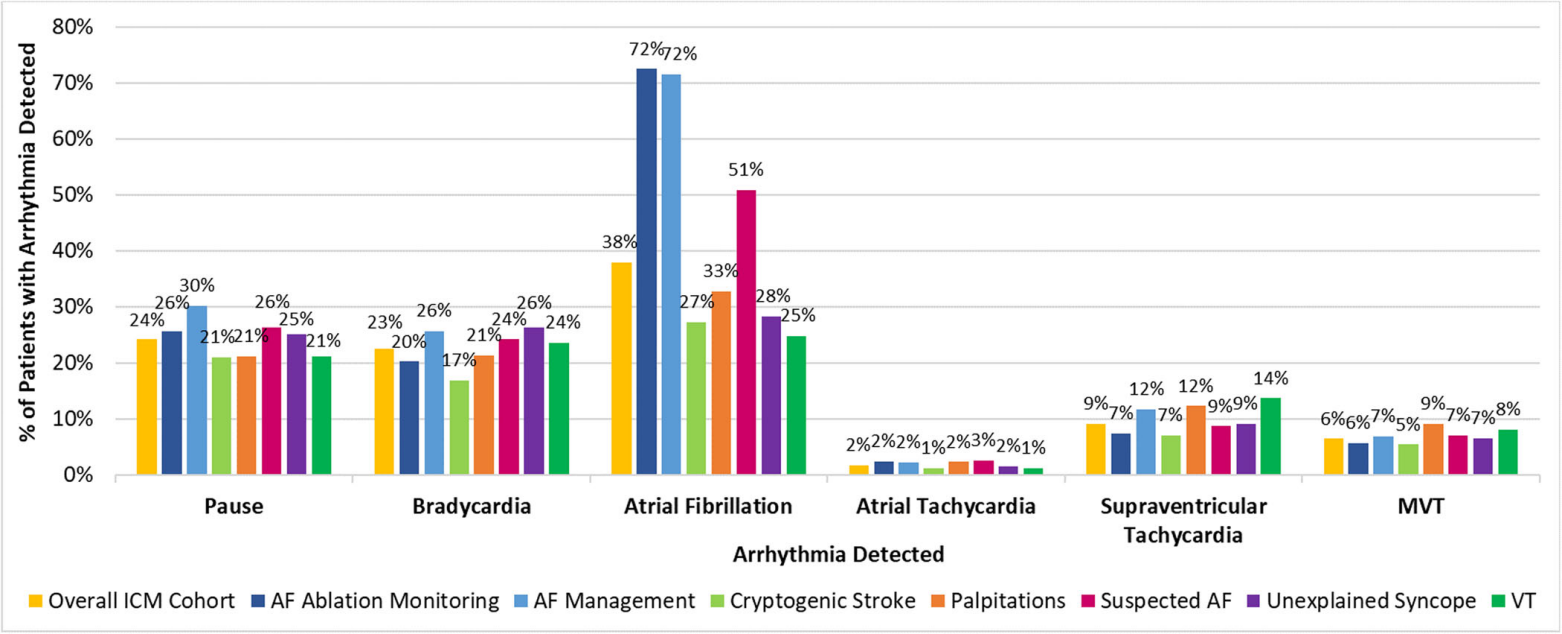
Types of arrhythmias detected by ICM, by patient indication for monitoring. AF, atrial fibrillation; ICM, insertable cardiac monitor; MVT, monomorphic ventricular tachycardia; VT, ventricular tachycardia. *Note:* Patients may contribute to more than one arrhythmia detection category, as all adjudicated ICM‐detected arrhythmias are represented for each patient. Detection rates for polymorphic ventricular tachycardia/ventricular fibrillation are not shown, as the percentages were < 1% for all patient groups aside from suspected AF (1.4%).

Of the 12 020 patients included in this analysis, 7284 (60.6%) had arrhythmias detected during the follow‐up period, with 3808 (31.68%) having one type of arrhythmia detected, 2203 (18.33%) having two types, and 1273 (10.6%) experiencing three or more distinct arrhythmia types (Figure 2). The most common was AF (37.9%), followed by pause (24.3%) and bradycardia (22.5%) (Figure 3). The mean (SD) time to the first, second, and last arrhythmia type detected was 7.1 (8.8), 11.6 (10.2), and 12.1 (10.6) months, respectively (Table S3).

##### 3.2.1.2 Suspected Arrhythmia Subgroup

In the 9847 patients receiving ICM for suspected arrhythmia, 5543 individuals (56.3%) had at least one detected arrhythmia during follow‐up. Within this group, 30.7% had one type of arrhythmia detected, 16.6% had two types, and 9.1% showed three or more types (Table S4). The most common arrhythmias in this subgroup were AF (30.5%), pause (23.2%), and bradycardia (22.0%). Notably, among patients receiving an ICM for unexplained syncope (*n* = 4015), 2357 (58.7%) had ≥ 1 arrhythmia detected, and of those 679 (28.8%) had pause or bradycardia alone detected, whereas the other 1678 (71.2%) had ≥ 1 arrhythmia *other than* pause/bradycardia detected during follow‐up. Additionally, in patients receiving ICM for cryptogenic stroke (*N* = 3769), 1900 (50.4%) had arrhythmias detected, and of those 1426 (75.1%) had ≥ 1 arrhythmia type *other than AF* detected (Table S4). A detailed breakdown of arrhythmias detected by indication for monitoring is presented in Figure 3.

The mean (SD) time to the first, second, and last arrhythmia type detected in this subgroup was 7.7 (9.0), 12.1 (10.2), and 12.4 (10.6) months, respectively (Table S3).

As reflected above, no arrhythmias were detected in 43.7% of patients with ICMs indicated for suspected arrhythmias. Closer examination reveals that no arrhythmias were identified in 42.1% of patients with ICMs for palpitations, or in 41.3% of patients monitored for unexplained syncope. Almost half (49.6%) of patients with ICMs for cryptogenic stroke did not have any arrhythmias identified over the follow‐up period. In a subgroup analysis of patients with ICMs for suspected arrhythmia patients with even longer (at least 2 years) follow‐up (*n* = 6962), 2902 (41.7%) had no detected arrhythmias.

##### 3.2.1.3 Management of Known AF Subgroup

In the 2173 patients receiving an ICM for management of known AF, 1741 (80.1%) had at least one detected arrhythmia, and among these, 1089 (62.6%) experienced one or more arrhythmias *that were not AF* (Table S4). The mean (SD) time to the first, second, and last arrhythmia types detected was 5.3 (7.7), 10.3 (9.9), and 11.2 (10.6), respectively (Table S3).

#### 3.2.2 Analysis #2: Therapeutic Interventions Analysis

##### 3.2.2.1 Total Interventions

**Figure 4:**
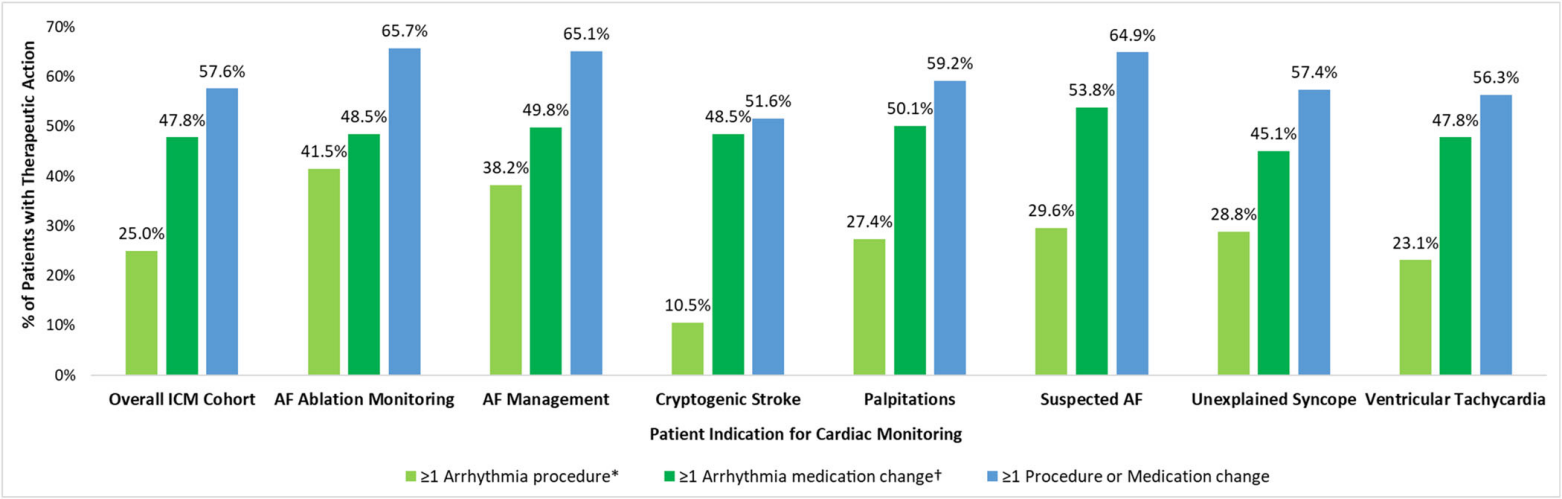
Cumulative arrhythmia‐related therapeutic interventions post‐ICM implant (procedures and medication changes), by patient indication. *Includes cardiovascular implantable electronic device implant or cardiac ablation. ^†^Includes initiation or discontinuation of arrhythmia‐related medication (antiarrhythmic drug, rate control medication, or oral anticoagulant).

Overall, 57.6% (*n* = 9820) of the 17 037 ICM patients received either an arrhythmia‐related procedure or a medication change (initiation or discontinuation) during post‐ICM implant follow‐up (Figure 4). Rates of individual interventions stratified by ICM indication are shown in Table 2 and summarized below.

**Table 2:** Therapeutic actions taken post‐ICM implant, by patient indication for monitoring.

ICM indication	Overall ICM cohort	Known AF indications		Suspected arrhythmia indications				
		AF Ablation	AF Mgmt	CS	Palpitations	Suspected AF	Unexplained Syncope	VT
Patients (*n*)	*N* = 17 037	431	2193	4852	1252	1346	6716	247
Arrhythmia‐related procedures, *n* (%)								
IPG implant	2404 (14.1%)	56 (13.0)	293 (13.4)	303 (6.2)	130 (10.4)	172 (12.8)	1435 (21.4)	15 (6.1)
ICD implant	251 (1.5%)	6 (1.4)	47 (2.1)	26 (0.5)	14 (1.1)	27 (2.0)	118 (1.8)	13 (5.3)
CRT implant	202 (1.2%)	6 (1.4)	52 (2.4)	16 (0.3)	9 (0.7)	19 (1.4)	97 (1.4)	3 (1.2)
Cardioversion	921 (5.4%)	84 (19.5)	331 (15.1)	86 (1.8)	60 (4.8)	119 (8.8)	234 (3.5)	7 (2.8)
Ablation^a^	1756 (10.3%)	120 (27.8)	515 (23.5)	192 (4.0)	220 (17.6)	205 (15.2)	472 (7.0)	32 (13.0)
Any CIEDs/ablations	3943 (23.1%)	155 (35.96%)	730 (33.3%)	472 (9.73%)	326 (26.0%)	352 (26.2%)	1854 (27.6%)	54 (21.9%)
Any CIEDs/ablations/cardioversions	4257 (25.0%)	179 (41.5%)	837 (38.2%)	509 (10.5%)	343 (27.4%)	398 (29.6%)	1934 (28.8%)	57 (23.1%)
Arrhythmia‐related medication changes								
OAC								
Initiation after ICM^b^	3894 (22.9%)	57 (13.2)	441 (20.1)	1505 (31.0)	268 (21.4)	364 (27.0)	1207 (18.0)	52 (21.1)
Use before ICM^c^	3077 (18.1%)	269 (62.4)	1038 (47.3)	282 (5.8)	170 (13.6)	430 (32.0)	858 (12.8)	30 (12.2)
Continuation after ICM^d^	2812 (16.5%)	253 (58.7)	940 (42.9)	252 (5.2)	162 (12.9)	370 (27.5)	806 (12.0)	29 (11.7)
Discontinuation^e^	724 (23.5%)	79 (29.4)	282 (27.2)	60 (21.3)	43 (25.3)	96 (22.3)	161 (18.8)	3 (10.0)
AAD								
Initiation after ICM	2352 (13.8%)	91 (21.1)	403 (18.4)	462 (9.5)	227 (18.1)	228 (16.9)	901 (13.4)	40 (16.2)
Use before ICM	1623 (9.5%)	123 (28.5)	611 (27.9)	79 (1.6)	138 (11.0)	234 (17.4)	406 (6.1)	32 (13.0)
Continuation after ICM	1300 (7.6%)	95 (22.0)	507 (23.1)	55 (1.1)	103 (8.2)	177 (13.2)	339 (5.1)	24 (9.7)
Discontinuation	652 (40.2%)	62 (50.4)	234 (38.3)	42 (53.2)	49 (35.5)	97 (41.5)	155 (38.2)	13 (40.6)
Rate control								
Initiation after ICM	4074 (23.9%)	84 (19.5)	456 (20.8)	1134 (23.4)	334 (26.7)	312 (23.2)	1691 (25.2)	63 (25.5)
Use before ICM	7399 (43.4%)	235 (54.5)	1174 (53.5)	1925 (39.7)	555 (44.3)	667 (49.6)	2730 (40.7)	113 (45.8)
Continuation after ICM	7006 (41.1%)	221 (51.3)	1121 (51.1)	1870 (38.5)	521 (41.6)	633 (47.0)	2531 (37.7)	109 (44.1)
Discontinuation	565 (7.6%)	33 (14.1)	132 (11.2)	107 (5.6)	38 (6.9)	54 (8.1)	195 (7.1)	6 (5.3)
Any medication initiation	7492 (44.0%)	173 (40.1%)	935 (42.6%)	2272 (46.8%)	576 (46.0%)	629 (46.7%)	2795 (41.6%)	112 (45.3%)
Any medication change (initiation or discontinuation)	8150 (47.8%)	209 (48.5%)	1092 (49.8%)	2351 (48.5%)	627 (50.1%)	724 (53.8%)	3029 (45.1%)	118 (47.8%)
Composite of procedures and medication changes								
Patients with any procedures or medication initiation	9284 (54.5%)	255 (59.2%)	1302 (59.4%)	2431 (50.1%)	701 (56.0%)	793 (58.9%)	3669 (54.6%)	133 (53.9%)
Patients with any procedures or medication change (initiation or discontinuation)	9820 (57.6%)	283 (65.7%)	1427 (65.1%)	2503 (51.6%)	741 (59.2%)	873 (64.9%)	3854 (57.4%)	139 (56.3%)

Abbreviations: AAD, antiarrhythmic drug; AF, atrial fibrillation; Cont., continuation; CRT, cardiac resynchronization therapy—with defibrillator (CRT‐D) or pacemaker (CRT‐P); CS, cryptogenic stroke; ICD, implantable cardioverter‐defibrillator; IPG, implantable pulse generator/pacemaker; Mgmt, management; OAC, Oral anticoagulation therapy; VT, ventricular tachycardia.

a Includes ablations for AF, supraventricular tachycardia, or VT.

^b^ Drug “initiation”: new claim for medication after ICM implant, with no claims during the 6 months before ICM implant.

^c^ Prior use: evidence of claims for medication up to 6 months before ICM implant.

^d^ Drug “continuation”: medication coverage after ICM implant, with evidence of claims during the 6 months before ICM implant as well.

^e^ Drug “discontinuation”: no claim during the last 100 days of follow‐up post‐ICM implant, but evidence of claims during the 6 months before ICM implant.

##### 3.2.2.2 Procedures

**Figure 5:**
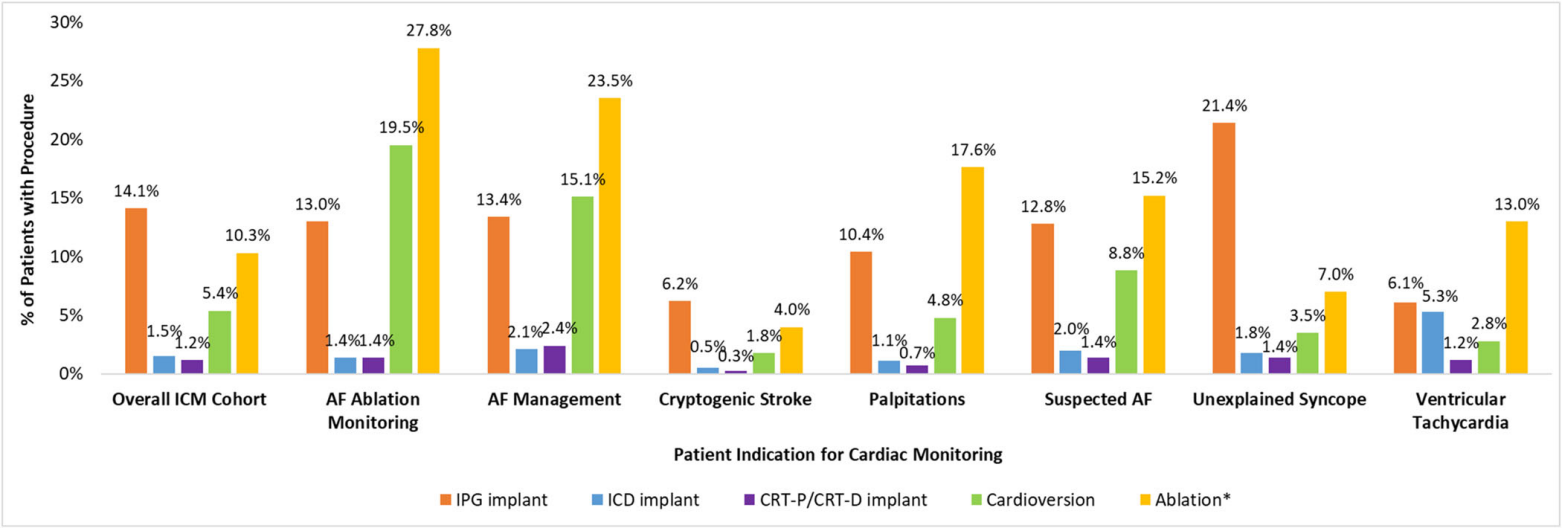
Arrhythmia‐related procedures received post‐ICM implant, by patient indication. AF, atrial fibrillation; CRT, cardiac resynchronization therapy‐ with defibrillator (CRT‐D) or pacemaker (CRT‐P); ICD, implantable cardioverter defibrillator; IPG, implantable pulse generator. *Includes ablations for AF, supraventricular tachycardia, or ventricular tachycardia.

One in four (25%) patients (*n* = 4257) in the full ICM cohort received a procedural intervention (CIED implant, ablation, or cardioversion); this equated to 23.1% (*n* = 3943) when cardioversions were excluded. Patients in the AF management and postablation AF monitoring categories had the highest rates of procedural interventions (38.2% and 41.5% respectively), with 23.5% of AF management patients and 27.8% of postablation AF monitoring patients undergoing catheter ablation during follow‐up (Figure 5). Ablation was also the most common procedural intervention in patients with ICMs indicated for palpitations (17.6%) and suspected AF (15.2%); overall, 19.7% of patients received an ablation at any time in the baseline or follow‐up periods, with 21.8% of these patients going on to receive at least one repeat ablation in follow‐up (Table S5). Implantable pulse generator (IPG) insertion was the most common procedure among ICM patients indicated for unexplained syncope (21.4%) and cryptogenic stroke (6.2%). When stratifying the ICM population between those with and without any history of heart failure (ICD‐10 diagnosis code I50.x), those with heart failure were more likely to receive all arrhythmia‐related procedures (Table S1).

##### 3.2.2.3 Medications

Among patients with post‐ICM interventions, 47.8% (*n* = 8150) received a medication change (initiation or discontinuation). Table 2 shows rates of medication changes by medication type (OAC, AAD, or rate control) stratified by ICM indication. Medication initiation rates for OACs were highest in the cryptogenic stroke subgroup (31.0% of patients); initiation of AAD was highest in the known AF subgroup (21.1% in postablation AF monitoring, 18.4% in AF management patients); and rate control medication initiation was highest in the palpitations category (26.7% of patients). Medication discontinuation rates for OACs were highest in the known AF population (29.1% in postablation AF monitoring, 27.2% in AF management patients); AAD discontinuation was highest in the cryptogenic stroke subgroup (53.2% of patients), followed closely by the postablation AF monitoring subgroup (50.4%); and for rate control medications, the highest discontinuation was observed in the known AF population (14.1%, in postablation AF monitoring, 11.2% in AF management patients).

##### 3.2.2.4 Time to Interventions

The mean (SD) time from ICM insertion to procedural intervention in the overall cohort was 13 (13) months (Table 3). Of the patients who received each type of procedure, the mean time from ICM insertion to the procedure was 13 (13) months for IPG implant, 18 (16) months for ICD (implantable cardioverter defibrillator) implant, 13 (13) months for CRT (cardiac resynchronization therapy) implant, 18 (16) months for cardioversion and 15 (14) months for ablation. Stratified by indication, the longest time to first procedural intervention was variable, ranging from 16 (14) months in the cryptogenic stroke category and 15 (14) months in the suspected AF category, to 12 (13) months in the palpitations category. Among the subgroup of patients with known AF, the time to first procedural intervention was 13 (13) months, consistent with the other categories reported above.

**Table 3:** Time from ICM implant to therapy.

Therapy type	Overall ICM cohort	Known AF indications		Suspected arrhythmia indications				
		AF ablation	AF Mgmt	CS	Palpitations	Suspected AF	Unexplained syncope	VT
Mean(SD) time to any first procedure, months^a^	13 (13)	13 (13)	13 (13)	16 (14)	12 (13)	15 (14)	12 (13)	14 (14)
IPG implant	13 (13)	19 (15)	16 (14)	17 (14)	14 (13)	17 (15)	11 (12)	15 (12)
ICD implant	18 (16)	18 (16)	22 (16)	25 (17)	13 (14)	19 (12)	17 (17)	14 (10)
CRT implant	13 (13)	18 (15)	16 (14)	17 (14)	13 (12)	17 (14)	11 (13)	14 (11)
Cardioversion	18 (16)	14 (14)	16 (15)	19 (15)	19 (16)	20 (18)	20 (16)	26 (24)
Ablation	15 (14)	17 (16)	13 (12)	17 (15)	13 (12)	13 (12)	17 (16)	15 (17)
Mean(SD) time to any first drug initiation, months^b^	7 (11)	3 (7)	4 (9)	7 (11)	8 (12)	5 (9)	9 (13)	8 (11)
OAC	13 (14)	8 (12)	11 (12)	11 (12)	16 (15)	12 (14)	16 (16)	14 (15)
AAD	16 (15)	10 (12)	13 (14)	18 (16)	16 (17)	16 (16)	16 (15)	10 (13)
Rate‐Control	14 (14)	13 (12)	14 (13)	12 (13)	15 (14)	14 (14)	15 (15)	13 (13)

Abbreviations: AF, atrial fibrillation; CS, cryptogenic stroke; Mgmt, management; SD, standard deviation; VT, ventricular tachycardia.

a Patients could have more than one procedure. Median [IQR] corresponds to the time to each type of procedure.

^b^ Patients could have initiated more than one type of drug. Median [IQR] corresponds to the time to initiation for each type of drug.

The mean time from ICM insertion to medication initiation was 7 (11) months in the full cohort, but was much shorter in the known AF subgroup, with mean time to medication initiation of 3 (7) months in the postablation AF monitoring category and 4 (9) months in the AF management category. Of the patients who initiated medication, the time to OAC initiation was 13 (14) months, 16 (15) months for AAD initiation, and 14 (14) months for rate‐control medication initiation. Based on the ICM indication category, time to initiation of medications post‐ICM insertion was variable in the suspected arrhythmias subgroup, ranging from 7 (11) months in cryptogenic stroke patients to 9 (13) months in patients with unexplained syncope.

## 4 Discussion

### 4.1 Arrhythmia Detection by ICMs

Small single‐center and registry studies of the diagnostic yield of ICMs in real‐world clinical practice have yielded disparate results, with diagnostic yields of 20%–70% reported in selected and unselected patient populations [24, 25, 26]. In this paper, we report the results from the largest‐to‐date analysis of real‐world data, which assessed the diagnostic yield of ICMs using a single ICM platform with previously validated AI‐enhanced diagnostic algorithms, and observed that more than 60% of patients implanted with ICMs for guideline‐approved clinical indications had ≥ 1 type of arrhythmia detected with robust (> 12 months) follow‐up. AF was the most common arrhythmia detected in this clinically heterogeneous population, an expected reflection of the patient demographic of older age and high prevalence of medical comorbidities such as hypertension, ischemic stroke, coronary artery disease (CAD), and diabetes mellitus.

We also found that ICMs were able to capture multiple types of arrhythmias, including incidental arrhythmias beyond those expected based on the clinical indication for monitoring, with more than 18% of the patients having at least two different types of arrhythmias and more than 10% having at least three types of arrhythmias during long‐term ICM monitoring. This high arrhythmic burden would likely have been undiagnosed or underdiagnosed in the absence of continuous long‐term monitoring with an ICM, and can provide clinicians with credible, comprehensive information to guide appropriate management strategies based on both the clinical indication and the arrhythmias detected.

Our analysis also shows that the time to detection of the first arrhythmia post‐ICM insertion, regardless of clinical indication, needs several months of monitoring and ranges from a mean (SD) duration of 6.3 (8.1) months for suspected AF to 8.4 (9.2) months for cryptogenic stroke. In addition, the time to detection between one type of arrhythmia and another also has a large degree of variability, with a mean (SD) duration of 7.4 (8.3) months between the detection of the first and second type of arrhythmia in the entire study cohort. Even in patients with known AF, the mean (SD) time to detection of the first arrhythmia was 5.3 (7.7) months post‐insertion, with the time between detection of the first and second arrhythmia in this subgroup of 7.1 (8.2) months. These observations reflect a potential benefit of long‐term continuous arrhythmia monitoring using ICMs both in patients with and without a history of prior arrhythmias, and mirror the high yield of continuous rhythm monitoring observed in multiple real‐world clinical settings [1, 2, 3].

In the unexplained syncope category, we found that pauses, bradycardias, and AF were seen at similar rates (25.2%, 26.4%, and 28.3% respectively). This finding expands upon previous smaller‐scale studies and meta‐analyses about the important incidence of tachycardias in this population beyond the expected yield of bradycardia and pauses, with slightly higher yields than previously demonstrated [27]. This suggests that many patients with unexplained syncope also have arrhythmias that may or may not be related to their symptoms, especially in older patients or those with other predisposing disease conditions like hypertension (HTN) or congestive heart failure (CHF) [28, 29].

In patients with cryptogenic stroke, AF was detected in 27%, and almost 51% of the patients in the suspected AF category were diagnosed with AF. This is consistent with findings from previous studies in clinical settings [4, 30]. However, we report the novel finding that 62.6% of patients with known AF (with ICMs for AF management or postablation AF monitoring) had arrhythmias *other than AF* detected by ICMs on long‐term monitoring, which highlights the high arrhythmic burden in this subgroup that may have been underdiagnosed or missed in the absence of continuous monitoring. Pauses (29.3%) and bradycardia (24.7%) were commonly identified in this subgroup of patients with known AF, possibly reflecting the impact of concomitant presence of sinus node dysfunction (tachy‐brady syndrome) or the use of rate‐control and certain rhythm‐control medications. In fact, while it was known that permanent AF combined with HF increases the odds of developing bradycardia [31], it has been recently observed that even in paroxysmal AF patients, concomitant bradyarrhythmias are not uncommon [32]. Based on our results from real‐world data, ICMs are helpful to reveal the presence of tachy‐brady syndrome in patients with known AF, and their identification may impact treatment decisions—such as avoiding certain medications (e.g., sotalol), or choosing a CIED‐supported strategy rather than AF ablation for procedural management [33].

Another clinically important role of ICMs implanted for suspected arrhythmias is their ability to credibly rule out an arrhythmic etiology (arrhythmia rule‐out). In our analysis, > 40% of patients with ICMs for suspected arrhythmias did not have any arrhythmias identified on long‐term monitoring, regardless of clinical indication. This included patients with ICMs for unexplained syncope (41.3%) and palpitations (42.1%)—two patient populations in whom making an etiologic diagnosis can be clinically challenging, and symptoms and the burden of multiple clinical tests can adversely impact patients' quality of life [34] and lead to significant healthcare utilization/costs [35, 36].

### 4.2 Therapeutic Interventions Following ICM Monitoring

This is the largest analysis exploring the rates of therapeutic interventions in patients with guideline‐approved clinical indications for ICM implantation with long‐term, continuous arrhythmia monitoring in a real‐world setting—reflecting contemporary clinical practice patterns for both medical and procedural management of these patients. We found that 57.6% of the patients included in this analysis received a therapeutic action post‐ICM insertion, with 25% receiving a procedural intervention and > 50% having undergone a medication change during follow‐up.

The observed rates of individual therapy types (medical and procedural) are in line with or greater than prior studies of ICM patients—with a higher cumulative therapy rate—likely because our analysis allowed for the inclusion of all relevant therapies compared to previous studies with more targeted endpoints [5, 35, 37, 38]. The observation of generally higher therapy rates could also be due to improvements in adherence to guideline recommendations and clinical risk stratification, leading to better patient selection [39]. For instance, the initiation rate for OACs in the cryptogenic stroke subgroup in the therapeutic interventions analysis was 31%—in alignment with the diagnostic yield of AF (27.2%) seen in the cryptogenic stroke subgroup in the arrhythmia detection analysis. These rates appear consistent with the high uptake of therapy post‐AF detection in this patient subgroup seen in other recent studies [3, 4].

Our analysis provides some interesting insights about practice patterns for the management of AF. In the subgroup of patients with ICM insertions for management of known AF, 27%–29% of patients had OAC discontinued post‐ICM, which is a real‐world representation of an approach proposed in some studies [40, 41], but not currently favored, especially for AF patients with high CHA_2_DS_2_‐VASc scores. In addition, in the postablation AF monitoring category, 50.4% of patients discontinued antiarrhythmics post‐ICM insertion, and 38.3% of patients with ICMs for AF management discontinued antiarrhythmics during follow‐up. These results suggest that cardiology providers may be more comfortable discontinuing medications in certain AF patients, as long as there is continuous, reliable AF monitoring using ICMs. This is particularly relevant in younger, physically active AF patients with low or borderline thromboembolic risk who may not want to stay on OACs long‐term, or older patients who are vulnerable to side effects of antiarrhythmic medications. Procedural interventions were also undertaken in a significant portion of the known AF patients, with over one‐quarter undergoing at least one ablation during post‐ICM insertion follow‐up.

### 4.3 Limitations and Future Research

A limitation of the analysis is the use of AI models rather than manual clinical adjudication to assess the accuracy of arrhythmia alert data, as it would not have been feasible to manually adjudicate ECGs from our cohort of 12 020 patients with long‐term monitoring follow‐up. This will likely lead to a slight overestimation of the incidence of arrhythmia since the PPV of the models is < 100%. However, in validation studies, the algorithms have been shown to successfully minimize false positives while preserving a high level of sensitivity, with PPVs generally in the range of 90%–95% [19, 20, 21, 22]. Another limitation is that the therapeutic interventions analysis relies on US‐only administrative claims data, which can lack granularity and is dependent on the coding process for accuracy and completeness.

It is important to note that our analyses are inherently descriptive in nature, since the cohort studied for the arrhythmia detection analysis was a subset of the cohort used for the therapeutic interventions analysis, and the arrhythmia monitoring data from Carelink were not comprehensively linked to the Optum CDM claims data. That said, both cohorts were sampled from the same Carelink device registry for ICM implants during the same time period, and there were no significant differences in the baseline clinical characteristics of the patients in the two cohorts (Table 1). In addition, the temporal association between arrhythmia detection and therapeutic action, and the clinical appropriateness of the therapeutic interventions, could not be adequately assessed due to the lack of direct linkage between a patient's arrhythmia detection data to their claims data. This also prevented us from assessing the therapeutic rates specifically associated with secondary (i.e., incidental/unexpected) findings. Although we were unable to determine a correlation between arrhythmia detection and therapeutic interventions in ICM patients in this analysis, the large sample size and long follow‐up duration in both cohorts provide real‐world insights about the high arrhythmic burden as well as current clinical practice patterns for both medical and procedural therapies in patients who receive ICMs for guideline‐approved indications in the United States. Finally, our study focuses solely on patients with long‐term continuous monitoring and AI‐adjudicated cardiac arrhythmia findings; as such, it does not compare diagnostic or treatment rates with those of patients who did not undergo long‐term monitoring. However, several clinical studies have demonstrated significantly higher rates of arrhythmia diagnosis and therapy in various populations of patients with versus without ICM monitoring [4, 5, 6, 7, 8, 9, 10].

Future studies are needed to prospectively study the temporal correlation between device‐detected arrhythmias using ICMs and therapeutic interventions in patients with and without a history of cardiac arrhythmias. Information on patients with known AF is particularly needed, as the real‐world use of AF burden and other AF characteristics to guide medical management is poorly understood. Research is underway to better understand current clinical decision‐making related to AF detection data, and to characterize the clinical implications of monitoring disease progression using ICMs (via ongoing analyses from the DEFINE AFib study, https://clinicaltrials.gov/study/NCT04926857), with the goal of formulating an individualized management plan for each AF patient that optimizes clinical outcomes and minimizes risk.

## 5 Conclusions

In a large, retrospective, real‐world analysis of patients with AI‐adjudicated ICM monitoring, > 60% had arrhythmias detected during follow‐up, with ~30% patients having ≥ 2 arrhythmias detected. Post‐ICM insertion, 57.6% of patients underwent an intervention related to arrhythmia management, with 25% undergoing procedural interventions. Regardless of clinical indication, > 40% of patients with ICMs for suspected arrhythmias had no arrhythmias identified during long‐term follow‐up.

## Ethics Statement

The retrospective data set was assessed, relative to the Health Insurance Portability and Accountability Act of 1996 (HIPAA), by a third‐party compliance partner and deemed low risk of identification via expert determination.

## Consent

Since this was a noninterventional, retrospective, observational study utilizing deidentified data, informed consent was not required from the patient under an IRB exemption status. All aspects of this study were conducted in compliance with the Health Insurance Portability and Accountability Act of 1996 (HIPAA) regulations and the HIPAA Omnibus Rule of 2013.

## Conflicts of Interest

S.A.S. is a consultant, advisory board member, and on the speakers' bureau for Medtronic. S.R., S.S., V.R., A.P.R., S.M., M.D.M., and J.J.S. are employees of Medtronic. J.Z. is a former employee of Medtronic.

## Supporting information

Supplement ‐ Incidental Findings Analysis.

## Data Availability

Because of contractual arrangements between Optum and Medtronic Inc., the data and study materials cannot be made available to other researchers for purposes of reproducing the results or replicating the procedure.
