# Supplementary material for: A Large, Real‐World Cohort Analysis of Arrhythmia Detection and Therapeutic Interventions in Patients With Insertable Cardiac Monitors and Long‐Term Monitoring
Source: J Cardiovasc Electrophysiol. 2025 Dec 17;37(2):359–73. doi: 10.1111/jce.70214 (PMC12891769; doi:10.1111/jce.70214)
Supplement: Supplementary file 1 — Supplement ‐ Incidental Findings Analysis. [file JCE-37-359-s001.docx]

**Supplemental Tables**

**Supplemental Table 1**. Claims data procedure codes

**Supplemental Table 2.** Additional baseline characteristics in arrhythmia detection and therapeutic intervention analyses

**Supplemental Table 3**. Time from ICM insertion to arrhythmia detection, by patient indication for monitoring

Supplemental Table 4. Number and types of AI-adjudicated arrhythmias detected, by patient indication for monitoring

**Supplemental Table 5**. Cardiac ablation procedures inclusive of pre-ICM baseline and post-ICM follow-up periods

**Supplemental Table 6**. Arrhythmia-related procedure rates in ICM patients with versus without a history of heart failure

**Supplemental Table 1.** Claims data procedure codes

| **Type** | **Code** | **Code Type** | **Code Description** |
| --- | --- | --- | --- |
| Cardioversion | 92960 | CPT Code | Cardioversion, elective, electrical conversion of arrhythmia; external |
| Cardioversion | 92961 | CPT Code | Cardioversion, elective, electrical conversion of arrhythmia; internal (separate procedure) |
| Cardioversion | 5A2204Z | ICD-10 PCS Code | Restoration of Cardiac Rhythm, Single |
| Cardioversion | 99.61 | ICD-9- PCS Code | Atrial cardioversion |
| Cardioversion | 99.62 | ICD-9- PCS Code | Other electric countershock of heart |
| Cardioversion | 37.91 | ICD-9- PCS Code | Open chest cardiac massage |
| Ablation | 93650 | CPT Code | Intracardiac catheter ablation of atrioventricular node function, atrioventricular conduction for creation of complete heart block, with or without temporary pacemaker placement |
| Ablation | 93651 | CPT Code | *(Note: code used through 2012):* Intracardiac catheter ablation of arrhythmogenic focus; for treatment of supraventricular tachycardia by ablation of fast or slow atrioventricular pathways, accessory atrioventricular connections or other atrial foci, singly or in combination |
| Ablation | 93653 | CPT Code | Comprehensive electrophysiologic evaluation with insertion and repositioning of multiple electrode catheters, induction or attempted induction of an arrhythmia with right atrial pacing and recording and catheter ablation of arrhythmogenic focus, including intracardiac electrophysiologic 3-dimensional mapping, right ventricular pacing and recording, left atrial pacing and recording from coronary sinus or left atrium, and His bundle recording, when performed; with treatment of supraventricular tachycardia by ablation of fast or slow atrioventricular pathway, accessory atrioventricular connection, cavo-tricuspid isthmus or other single atrial focus or source of atrial re-entry |
| Ablation | 93654 | CPT Code | Comprehensive electrophysiologic evaluation with insertion and repositioning of multiple electrode catheters, induction or attempted induction of an arrhythmia with right atrial pacing and recording and catheter ablation of arrhythmogenic focus, including intracardiac electrophysiologic 3-dimensional mapping, right ventricular pacing and recording, left atrial pacing and recording from coronary sinus or left atrium, and His bundle recording, when performed; with treatment of ventricular tachycardia or focus of ventricular ectopy including left ventricular pacing and recording, when performed |
| Ablation | 93656 | CPT Code | Comprehensive electrophysiologic evaluation including transseptal catheterizations, insertion and repositioning of multiple electrode catheters with intracardiac catheter ablation of AF by pulmonary vein isolation, including intracardiac electrophysiologic 3-dimensional mapping, intracardiac echocardiography including imaging supervision and interpretation, induction or attempted induction of an arrhythmia including left or right atrial pacing/recording, right ventricular pacing/recording, and His bundle recording when performed |
| Ablation | 93655 | CPT Code | Intracardiac catheter ablation of a discrete mechanism of arrhythmia which is distinct from the primary ablated mechanism, including repeat diagnostic maneuvers, to treat a spontaneous or induced arrhythmia |
| Ablation | 93657 | CPT Code | Additional linear or focal intracardiac catheter ablation of the left or right atrium for treatment of atrial fibrillation remaining after completion of pulmonary vein isolation |
| Ablation | 33250 | CPT Code | Operative ablation of supraventricular arrhythmogenic focus or pathway (eg, Wolff-Parkinson-White, atrioventricular node re-entry), tract(s) and/or focus (foci); without cardiopulmonary bypass |
| Ablation | 33251 | CPT Code | Operative ablation of supraventricular arrhythmogenic focus or pathway (eg, Wolff-Parkinson-White, atrioventricular node re-entry), tract(s) and/or focus (foci); with cardiopulmonary bypass |
| Ablation | 33254 | CPT Code | Operative tissue ablation and reconstruction of atria, limited (eg, modified maze procedure) |
| Ablation | 33255 | CPT Code | Operative tissue ablation and reconstruction of atria, extensive (eg, maze procedure); without cardiopulmonary bypass |
| Ablation | 33256 | CPT Code | Operative tissue ablation and reconstruction of atria, extensive (eg, maze procedure); with cardiopulmonary bypass |
| Ablation | 33257 | CPT Code | Operative tissue ablation and reconstruction of atria, performed at the time of other cardiac procedure(s), limited (eg, modified maze procedure) (List separately in addition to code for primary procedure) |
| Ablation | 33258 | CPT Code | Operative tissue ablation and reconstruction of atria, performed at the time of other cardiac procedure(s), extensive (eg, maze procedure), without cardiopulmonary bypass (List separately in addition to code for primary procedure) |
| Ablation | 33259 | CPT Code | Operative tissue ablation and reconstruction of atria, performed at the time of other cardiac procedure(s), extensive (eg, maze procedure), with cardiopulmonary bypass (List separately in addition to code for primary procedure) |
| Ablation | 33261 | CPT Code | Operative ablation of ventricular arrhythmogenic focus with cardiopulmonary bypass |
| Ablation | 37.34 | ICD-9-PCS Code | Excision or destruction of other lesion or tissue of heart, endovascular approach |
| Ablation | 37.33 | ICD-9-PCS Code | Excision or destruction of other lesion or tissue of heart, open approach |
| Ablation | 02583ZZ | ICD-10-PCS Code | Destruction of conduction mechanism, percutaneous approach |
| Ablation | 4A0234Z | ICD-10-PCS Code | Measurement of cardiac electrical activity, percutaneous approach |
| Ablation | 025K0ZZ | ICD-10-PCS Code | Destruction of Right Ventricle, Open Approach |
| Ablation | 025L0ZZ | ICD-10-PCS Code | Destruction of Left Ventricle, Open Approach |
| Ablation | 02550ZZ | ICD-10-PCS Code | Destruction of Atrial Septum, Open Approach |
| Ablation | 02560ZZ | ICD-10-PCS Code | Destruction of Right Atrium, Open Approach |
| Ablation | 02570ZK | ICD-10-PCS Code | Destruction of Left Atrial Appendage, Open Approach |
| Ablation | 02570ZZ | ICD-10-PCS Code | Destruction of Left Atrium, Open Approach |
| Ablation | 02580ZZ | ICD-10-PCS Code | Destruction of Conduction Mechanism, Open Approach |
| Ablation | 02590ZZ | ICD-10-PCS Code | Destruction of Chordae Tendineae, Open Approach |
| Ablation | 025M0ZZ | ICD-10-PCS Code | Destruction of Ventricular Septum, Open Approach |
| Ablation | 5A1221J | ICD-10-PCS Code | Performance of Cardiac Output, Continuous, Automated |
| Ablation | 5A1221Z | ICD-10-PCS Code | Performance of Cardiac Output, Continuous |
| Pacemaker | 33206 | CPT Code | Insertion of new or replacement of permanent pacemaker with transvenous electrode(s); atrial |
| Pacemaker | 33207 | CPT Code | Insertion of new or replacement of permanent pacemaker with transvenous electrode(s); ventricular |
| Pacemaker | 33208 | CPT Code | Insertion of new or replacement of permanent pacemaker with transvenous electrode(s); atrial and ventricular |
| Pacemaker | 33212 | CPT Code | Insertion of pacemaker pulse generator only; with existing single lead |
| Pacemaker | 33213 | CPT Code | Insertion of pacemaker pulse generator only; with existing dual leads |
| Pacemaker | 33221 | CPT Code | Insertion of pacemaker pulse generator only; with existing multiple leads |
| Pacemaker | 33214 | CPT Code | Upgrade of implanted pacemaker system, conversion of single chamber system to dual chamber system (includes removal of previously placed pulse generator, testing of existing lead, insertion of new lead, insertion of new pulse generator) |
| Pacemaker | 33274 | CPT Code | Transcatheter insertion or replacement of permanent leadless pacemaker, right ventricular, including imaging guidance (e.g., fluoroscopy, venous ultrasound, ventriculography, femoral venography) and device evaluation (e.g., interrogation or programming), when performed |
| Pacemaker | 33227 | CPT Code | Removal of permanent pacemaker pulse generator with replacement of pacemaker pulse generator; single lead system |
| Pacemaker | 33228 | CPT Code | Removal of permanent pacemaker pulse generator with replacement of pacemaker pulse generator; dual lead system |
| Pacemaker | 33229 | CPT Code | Removal of permanent pacemaker pulse generator with replacement of pacemaker pulse generator; multiple lead system |
| Pacemaker | 0387T | CPT Code | Transcatheter insertion or replacement of permanent leadless pacemaker, ventricular |
| Pacemaker | 0JH606Z | ICD-10 PCS Code | Insertion of pacemaker, dual chamber into chest subcutaneous tissue and fascia, open approach |
| Pacemaker | 0JH806Z | ICD-10 PCS Code | Insertion of pacemaker, dual chamber into abdomen subcutaneous tissue and fascia, open approach |
| Pacemaker | 0JH605Z | ICD-10 PCS Code | Insertion of pacemaker, single chamber rate responsive into chest subcutaneous tissue and fascia, open approach |
| Pacemaker | 0JH604Z | ICD-10 PCS Code | Insertion of pacemaker, single chamber into chest subcutaneous tissue and fascia, open approach |
| Pacemaker | 0JH804Z | ICD-10 PCS Code | Insertion of pacemaker, single chamber into abdomen subcutaneous tissue and fascia, open approach |
| Pacemaker | 0JH805Z | ICD-10 PCS Code | Insertion of pacemaker, single chamber rate responsive into abdomen subcutaneous tissue and fascia, open approach |
| Pacemaker | 02HK3NZ | ICD-10 PCS Code | Insertion of intracardiac pacemaker into right ventricle, percutaneous approach |
| Pacemaker | 02WA3NZ | ICD-10 PCS Code | Revision of intracardiac pacemaker in heart, percutaneous approach |
| Pacemaker | 0JH636Z | ICD-10 PCS Code | Insertion of pacemaker, dual chamber into chest subcutaneous tissue and fascia, percutaneous approach |
| Pacemaker | 0JH6836Z | ICD-10 PCS Code | Insertion of pacemaker, dual chamber into abdomen subcutaneous tissue and fascia, percutaneous approach |
| Pacemaker | 0JH635Z | ICD-10 PCS Code | Insertion of pacemaker, single chamber rate responsive into chest subcutaneous tissue and fascia, percutaneous approach |
| Pacemaker | 0JH634Z | ICD-10 PCS Code | Insertion of pacemaker, single chamber into chest subcutaneous tissue and fascia, percutaneous approach |
| Pacemaker | 0JH834Z | ICD-10 PCS Code | Insertion of pacemaker, single chamber into abdomen subcutaneous tissue and fascia, percutaneous approach |
| Pacemaker | 0JH835Z | ICD-10 PCS Code | Insertion of pacemaker, single chamber rate responsive into abdomen subcutaneous tissue and fascia, percutaneous approach |
| Pacemaker | 34.85 | ICD-9-PCS Code | Implantation of diaphragmatic pacemaker |
| Pacemaker | 37.8 | ICD-9-PCS Code | Insertion of permanent pacemaker, initial or replacement, type of device not specified |
| Pacemaker | 37.81 | ICD-9-PCS Code | Initial insertion of single-chamber device, not specified as rate responsive |
| Pacemaker | 37.82 | ICD-9-PCS Code | Initial insertion of single-chamber device, rate responsive |
| Pacemaker | 37.83 | ICD-9-PCS Code | Initial insertion of dual-chamber device |
| Pacemaker | 37.86 | ICD-9-PCS Code | Replacement of any type of pacemaker device with single-chamber device, rate responsive |
| Pacemaker | 37.87 | ICD-9-PCS Code | Insertion of Pacemaker, Dual Chamber into Chest Subcutaneous Tissue and Fascia, Open Approach |
| Pacemaker | 37.89 | ICD-9-PCS Code | Revision or removal of pacemaker device |
| CRT-P | 33206 and 33225 | CPT Code | Insertion of new or replacement of permanent pacemaker with transvenous electrode(s); atrial  **AND**  Insertion of pacing electrode, cardiac venous system, for left ventricular pacing, at time of insertion of implantable defibrillator or pacemaker pulse generator (eg, for upgrade to dual chamber system) |
| CRT-P | 33207 and 33225 | CPT Code | Insertion of new or replacement of permanent pacemaker with transvenous electrode(s); ventricular  **AND**  Insertion of pacing electrode, cardiac venous system, for left ventricular pacing, at time of insertion of implantable defibrillator or pacemaker pulse generator (eg, for upgrade to dual chamber system) |
| CRT-P | 33208 and 33225 | CPT Code | Insertion of new or replacement of permanent pacemaker with transvenous electrode(s); atrial and ventricular  **AND**  Insertion of pacing electrode, cardiac venous system, for left ventricular pacing, at time of insertion of implantable defibrillator or pacemaker pulse generator (eg, for upgrade to dual chamber system) |
| CRT-P | 33228 | CPT Code | Removal of permanent pacemaker pulse generator with replacement of pacemaker pulse generator; dual lead system |
| CRT-P | 33229 | CPT Code | Removal of permanent pacemaker pulse generator with replacement of pacemaker pulse generator; multiple lead system |
| CRT-P | 0JH607Z | ICD-10 PCS Code | Insertion of cardiac resynchronization pacemaker pulse generator into chest subcutaneous tissue and  fascia, open approach |
| CRT-P | 0JH807Z | ICD-10 PCS Code | Insertion of cardiac resynchronization pacemaker pulse generator into abdomen subcutaneous tissue  and fascia, open approach |
| CRT-P | 02H43JZ | ICD-10 PCS Code | Insertion of pacemaker lead into coronary vein, percutaneous approach |
| CRT-P | 0JH637Z | ICD-10 PCS Code | Insertion of cardiac resynchronization pacemaker pulse generator into chest subcutaneous tissue and  fascia, percutaneous approach |
| CRT-P | 0JH837Z | ICD-10 PCS Code | Insertion of cardiac resynchronization pacemaker pulse generator into abdomen subcutaneous tissue  and fascia, percutaneous approach |
| CRT-P | 0.5 | ICD-9-PCS Code | Implantation of cardiac resynchronization pacemaker without mention of defibrillation, total system (CRT-P) |
| CRT-P | 0.53 | ICD-9-PCS Code | Implantation or replacement of cardiac resynchronization pacemaker pulse generator only (CRT-P) |
| CRT-D | 33249 and 33225 | CPT Code | Insertion or replacement of permanent implantable defibrillator system, with transvenous lead(s),  single or dual chamber  **AND** Insertion of LV pacing electrode, at time of insertion ICD or pacemaker pulse generator (list separately in addition to code for primary procedure) |
| CRT-D | 33262 | CPT Code | Remove and replace implantable defibrillator generator; single lead system |
| CRT-D | 33263 | CPT Code | Remove and replace implantable defibrillator generator; dual lead system |
| CRT-D | 33264 | CPT Code | Removal of implantable defibrillator pulse generator with replacement of implantable defibrillator pulse generator; multiple lead system |
| CRT-D | 0JH609Z | ICD-10 PCS Code | Insertion of cardiac resynchronization defibrillator pulse generator into chest subcutaneous tissue  and fascia, open approach |
| CRT-D | 0JH809Z | ICD-10 PCS Code | Insertion of cardiac resynchronization defibrillator pulse generator into abdomen subcutaneous  tissue and fascia, open approach |
| CRT-D | 02H43KZ | ICD-10 PCS Code | Insertion of defibrillator lead into coronary vein, percutaneous approach |
| CRT-D | 0JH639Z | ICD-10 PCS Code | Insertion of cardiac resynchronization defibrillator pulse generator into chest subcutaneous tissue  and fascia, percutaneous approach |
| CRT-D | 0JH839Z | ICD-10 PCS Code | Insertion of cardiac resynchronization defibrillator pulse generator into abdomen subcutaneous  tissue and fascia, percutaneous approach |
| CRT-D | 0.51 | ICD-9-PCS Code | Implantation of cardiac resynchronization defibrillator, total system (CRT-D) |
| CRT-D | 0.54 | ICD-9-PCS Code | Implantation or replacement of cardiac resynchronization defibrillator pulse generator device only (CRT-D) |
| ICD | 33249 | CPT Code | Insertion or replacement of permanent implantable defibrillator system, with transvenous lead(s),  single or dual chamber |
| ICD | 33230 | CPT Code | Insert implantable defibrillator generator only; with existing dual leads |
| ICD | 33231 | CPT Code | Insert implantable defibrillator generator only; with existing multiple leads |
| ICD | 33240 | CPT Code | Insert implantable defibrillator generator only; with existing single lead |
| ICD | 33262 | CPT Code | Remove and replace implantable defibrillator generator; single lead system |
| ICD | 33263 | CPT Code | Remove and replace implantable defibrillator generator; dual lead system |
| ICD | 33264 | CPT Code | Removal of implantable defibrillator pulse generator with replacement of implantable defibrillator pulse generator; multiple lead system |
| ICD | 0JH608Z | ICD-10 PCS Code | Insertion of defibrillator generator into chest subcutaneous tissue and fascia, open approach |
| ICD | 0JH638Z | ICD-10 PCS Code | Insertion of defibrillator generator into chest subcutaneous tissue and fascia, percutaneous approach |
| ICD | 0JH808Z | ICD-10 PCS Code | Insertion of defibrillator generator into abdomen subcutaneous tissue and fascia, open approach |
| ICD | 0JH838Z | ICD-10 PCS Code | Insertion of defibrillator generator into abdomen subcutaneous tissue and fascia, percutaneous approach |
| ICD | 0JH60PZ | ICD-10 PCS Code | Insertion of Cardiac Rhythm Related Device into Chest Subcutaneous Tissue and Fascia, Open Approach |
| ICD | 0JH63PZ | ICD-10 PCS Code | Insertion of Cardiac Rhythm Related Device into Chest Subcutaneous Tissue and Fascia, percutaneous Approach |
| ICD | 0.52 | ICD-9-PCS Code | Implantation or replacement of transvenous lead [electrode] into left ventricular coronary venous system |
| ICD | 37.94 | ICD-9-PCS Code | Implantation or replacement of automatic cardioverter/defibrillator, total system [AICD] |
| ICD | 37.96 | ICD-9-PCS Code | Implantation of automatic cardioverter/ defibrillator pulse generator only |
| LAA Closure | 33340 | CPT Code | Percutaneous transcatheter closure of the left atrial appendage with endocardial implant, including fluoroscopy, transseptal puncture, catheter placement, left atrial angiography, left atrial appendage angiography, when performed, and radiological supervision and interpretation |
| LAA Closure | 02L73DK | ICD-10 PCS Code | Occlusion of left atrial appendage with intraluminal device, percutaneous approach |

Abbreviations: CPT, current procedural terminology; CRT, chronic resynchronization therapy; ICD, international classification of diseases; LAA, left atrial appendage.

**Supplemental Table 2.** Additional baseline characteristics in arrhythmia detection and therapeutic intervention analyses

|  | **Overall ICM Cohort** | **Known AF Indications** | | **Suspected Arrythmia Indications** | | | | |
| --- | --- | --- | --- | --- | --- | --- | --- | --- |
| **Analysis** |  | **AF Ablation Monitoring** | **AF Management** | **Cryptogenic Stroke** | **Palpitations** | **Suspected AF** | **Unexplained Syncope** | **Ventricular Tachycardia** |
| **Region** |  |  |  |  |  |  |  |  |
| Northeast | 1891 (15.7%) | 75 (18.3%) | 270 (15.3%) | 661 (17.5%) | 116 (12.2%) | 125 (13.1%) | 620 (15.4%) | 24 (14.9%) |
|  | 2707 (15.9%) | 74 (17.2%) | 360 (16.4%) | 870 (17.9%) | 168 (13.4%) | 188 (14.0%) | 1000 (14.9%) | 47 (19.0%) |
| South | 4775 (39.7%) | 139 (33.9%) | 710 (40.3%) | 1452 (38.5%) | 430 (45.3%) | 345 (36.2%) | 1636 (40.8%) | 63 (39.1%) |
|  | 7093 (41.6%) | 164 (38.1%) | 954 (43.5%) | 1942 (40.0%) | 578 (46.2%) | 505 (37.5%) | 2854 (42.5%) | 96 (38.9%) |
| Midwest | 2903 (24.2%) | 83 (20.2%) | 445 (25.2%) | 981 (26.0%) | 199 (21.0%) | 218 (22.9%) | 945 (23.5%) | 32 (19.9%) |
|  | 4018 (23.6%) | 95 (22.0%) | 525 (23.9%) | 1219 (25.1%) | 264 (21.1%) | 332 (24.7%) | 1540 (22.9%) | 43 (17.4%) |
| West | 1978 (16.5%) | 95 (23.2%) | 273 (15.5%) | 523 (13.9%) | 170 (17.9%) | 221 (23.2%) | 663 (16.5%) | 33 (20.5%) |
|  | 3209 (18.8%) | 98 (22.7%) | 352 (16.1%) | 820 (16.9%) | 240 (19.2%) | 321 (23.9%) | 1317 (19.6%) | 61 (24.7%) |
| Unknown | 473 (3.9%) | 18 (4.4%) | 65 (3.7%) | 152 (4.0%) | 35 (3.7%) | 43 (4.5%) | 151 (3.8%) | 9 (5.6%) |
|  | 10 (0.1%) | 0 | 2 (0.1%) | 1 (0%) | 2 (0.2%) | 0 | 5 (0.1%) | 0 |
| **Insurance type** |  |  |  |  |  |  |  |  |
| Commercial | 2598 (21.6%) | 133 (32.4%) | 423 (24.0%) | 738 (19.6%) | 331 (34.8%) | 180 (18.9%) | 730 (18.2%) | 63 (39.1%) |
|  | 3240 (19.0%) | 108 (25.1%) | 438 (20.0%) | 862 (17.8%) | 405 (32.4%) | 226 (16.8%) | 1109 (16.5%) | 92 (37.2%) |
| Medicare Advantage | 9422 (78.4%) | 277 (67.6%) | 1340 (76.0%) | 3031 (80.4%) | 619 (65.2%) | 772 (81.1%) | 3285 (81.8%) | 98 (60.1%) |
|  | 13797 (81.0%) | 323 (74.9%) | 1755 (80.0%) | 3990 (82.2%) | 847 (67.7%) | 1120 (83.2%) | 5607 (83.5%) | 155 (62.8%) |
| **Year of Implant** |  |  |  |  |  |  |  |  |
| 2016 | 515 (4.3%) | 25 (6.1%) | 68 (3.9%) | 129 (3.4%) | 58 (6.1%) | 39 (4.1%) | 190 (4.7%) | 6 (3.7%) |
|  | 550 (3.2%) | 0 | 0 | 0 | 95 (7.6%) | 55 (4.1%) | 389 (5.8%) | 11 (4.5%) |
| 2017 | 3114 (25.9%) | 112 (27.3%) | 400 (22.7%) | 923 (24.5%) | 282 (29.7%) | 234 (24.6%) | 1126 (28.0%) | 37 (23.0%) |
|  | 4031 (23.7%) | 100 (23.2%) | 438 (20.0%) | 964 (19.9%) | 329 (26.3%) | 317 (23.6%) | 1837 (27.4%) | 46 (18.6%) |
| 2018 | 4000 (33.3%) | 130 (31.7%) | 607 (34.4%) | 1265 (33.6%) | 315 (33.2%) | 299 (31.4%) | 1337 (33.3%) | 47 (29.2%) |
|  | 5003 (29.4%) | 146 (33.9%) | 681 (31.1%) | 1460 (30.1%) | 377 (30.1%) | 377 (28.0%) | 1894 (28.2%) | 68 (27.5%) |
| 2019 | 4390 (36.5%) | 143 (34.9%) | 688 (39.0%) | 1451 (38.5%) | 295 (31.1%) | 380 (39.9%) | 1362 (33.9%) | 71 (44.1%) |
|  | 5460 (32.1%) | 142 (33%) | 800 (36.5%) | 1725 (35.6%) | 322 (25.7%) | 448 (33.3%) | 1929 (28.7%) | 94 (38.1%) |
| 2020 | 1 (0.01%) | 0 | 0 | 1 (0.03%) | 0 | 0 | 0 | 0 |
|  | 1993 (11.7%) | 43 (10%) | 274 (12.5%) | 703 (14.5%) | 129 (10.3%) | 149 (11.1%) | 667 (9.9%) | 28 (11.3%) |

Abbreviations: AF, atrial fibrillation; ICM, insertable cardiac monitor.

**Supplemental Table 3**. Time from ICM insertion to arrhythmia detection, by patient indication for monitoring

|  |  | **Known AF Indications** | | | **Suspected Arrythmia Indications** | | | |
| --- | --- | --- | --- | --- | --- | --- | --- | --- |
|  | **Overall ICM cohort** | **AF Ablation Monitoring** | **AF Management** | **Cryptogenic Stroke** | **Palpitations** | **Suspected AF** | **Unexplained Syncope** | **Ventricular Tachycardia** |
| **Time from ICM implant to 1st arrhythmia type detected, months** |  |  |  |  |  |  |  |  |
| N | **7284** | 322 | 1419 | 1900 | 550 | 652 | 2357 | 84 |
| Mean (SD) | **7.07 (8.77)** | 5.42 (8.25) | 5.21 (7.53) | 8.37 (9.22) | 7.2 (9.15) | 6.27 (8.08) | 7.59 (8.98) | 6.54 (9.30) |
| Median [IQR] | **3 [1, 11]** | 1.5 [0, 8] | 2 [0, 7] | 5 [1, 13] | 3 [1, 11] | 3 [0, 10] | 4 [1, 12] | 2 [0, 8.50] |
| (Min, Max) | **(0, 44)** | (0, 41) | (0, 44) | (0, 43) | (0, 42) | (0, 44) | (0, 42) | (0, 41) |
| **Time from ICM implant to 2nd arrhythmia type detected, months** |  |  |  |  |  |  |  |  |
| n (%) with >1 arrhythmia diagnosed | **3476** | 162 | 792 | 782 | 267 | 337 | 1099 | 37 |
| Mean (SD) | **11.63 (10.17)** | 10.82 (10.67) | 10.24 (9.69) | 13.05 (10.22) | 11.75 (10.43) | 10.08 (9.95) | 12.25 (10.27) | 10.27 (8.30) |
| Median [IQR] | **9 [3, 18]** | 7 [2, 19] | 7 [2, 16] | 11 [4, 20] | 9 [3, 17] | 7 [2, 16] | 10 [3, 19] | 9 [4, 14] |
| (Min, Max) | **(0, 44)** | (0, 42) | (0, 44) | (0, 44) | (0, 43) | (0, 44) | (0, 43) | (0, 34) |
| **Time between 1st and 2nd arrhythmia type detected, months** |  |  |  |  |  |  |  |  |
| n (%) with >1 arrhythmia diagnosed | **3476** | 162 | 792 | 782 | 267 | 337 | 1099 | 37 |
| Mean (SD) | **7.44 (8.25)** | 7.56 (8.67) | 6.96 (8.16) | 7.93 (8.13) | 7.69 (8.74) | 6.76 (8.12) | 7.57 (8.26) | 7.76 (8.16) |
| Median [IQR] | **4 [1, 11]** | 4 [1, 12] | 4 [1, 10] | 5 [1, 12] | 5 [1, 12] | 4 [1, 10] | 4 [1, 12] | 6 [1, 12] |
| (Min, Max) | **(0, 42)** | (0, 38) | (0, 42) | (0, 40) | (0, 40) | (0, 41) | (0, 41) | (0, 32) |
| **Time from ICM implant to last arrhythmia type detected, months** |  |  |  |  |  |  |  |  |
| N | **7284** | 322 | 1419 | 1900 | 550 | 652 | 2357 | 84 |
| Mean (SD) | **12.14 (10.61)** | 10.62 (10.76) | 11.28 (10.58) | 12.72 (10.53) | 12.47 (11.11) | 11.4 (10.29) | 12.52 (10.55) | 11.88 (11.34) |
| Median [IQR] | **10 [3, 19]** | 7 [1, 17] | 8 [2, 18] | 10 [4, 20] | 9 [3, 20] | 9 [3, 18] | 11 [3, 20] | 10 [2, 18.50] |
| (Min, Max) | **(0, 46)** | (0, 42) | (0, 44) | (0, 44) | (0, 43) | (0, 44) | (0, 46) | (0, 43) |

Abbreviations: AF, atrial fibrillation; ICM, insertable cardiac monitor; IQR, interquartile range; SD, standard deviation.

Supplemental Table 4. Number **and types of AI-adjudicated arrhythmias detected, by patient indication for monitoring**

|  |  | **Known AF indications** | | **Suspected arrhythmia indications** | | | | |
| --- | --- | --- | --- | --- | --- | --- | --- | --- |
| **ICM indication** | **Overall ICM cohort** | **AF Ablation** | **AF Mgmt** | **CS** | **Palpitations** | **Suspected AF** | **Unexplained Syncope** | **VT** |
| **Patients (n)** | 12,020 | 410 | 1,763 | 3,769 | 950 | 952 | 4,015 | 161 |
| **Any arrhythmia detected, n (%)** | 7284 (60.60%) | 322 (78.5) | 1419 (80.5) | 1900 (50.4) | 550 (57.9) | 652 (68.5) | 2357 (58.7) | 84 (52.2) |
| Pause | 2924 (24.33%) | 105 (25.6) | 532 (30.2) | 790 (21.0) | 201 (21.2) | 251 (26.4) | 1011 (25.2) | 34 (21.1) |
| Bradycardia | 2704 (22.50%) | 83 (20.2) | 453 (25.7) | 638 (16.9) | 202 (21.3) | 230 (24.2) | 1060 (26.4) | 38 (23.6) |
| AF | 4555 (37.90%) | 297 (72.4) | 1260 (71.5) | 1025 (27.2) | 311 (32.7) | 484 (50.8) | 1138 (28.3) | 40 (24.8) |
| AT | 210 (1.75%) | 10 (2.4) | 39 (2.2) | 46 (1.2) | 22 (2.3) | 25 (2.6) | 66 (1.6) | 2 (1.2) |
| SVT | 1092 (9.08%) | 30 (7.3) | 207 (11.7) | 269 (7.1) | 117 (12.3) | 83 (8.7) | 364 (9.1) | 22 (13.7) |
| MVT | 776 (6.46%) | 23 (5.6) | 122 (6.9) | 202 (5.4) | 86 (9.1) | 68 (7.1) | 262 (6.5) | 13 (8.1) |
| PVT/VF | 82 (0.68%) | 3 (0.7) | 14 (0.8) | 23 (0.6) | 4 (0.4) | 13 (1.4) | 25 (0.6) | 0 |
| **Stratified by number of types of arrhythmias detected, n (%)** | | | | | | | | |
| 0 Arrhythmia | 4736 (39.40%) | 88 (21.5) | 344 (19.5) | 1869 (49.6) | 400 (42.1) | 300 (31.5) | 1658 (41.3) | 77 (47.8) |
| 1 Arrhythmia | 3808 (31.68%) | 160 (39.0) | 627 (35.6) | 1118 (29.7) | 283 (29.8) | 315 (33.1) | 1258 (31.3) | 47 (29.2) |
| Pause | 723 (6.01%) | 7 (1.7) | 33 (1.9) | 283 (7.5) | 49 (5.2) | 41 (4.3) | 300 (7.5) | 10 (6.2) |
| Bradycardia | 788 (6.56%) | 2 (0.5) | 59 (3.4) | 216 (5.7) | 63 (6.6) | 53 (5.6) | 379 (9.4) | 16 (9.9) |
| AF | 1889 (15.72%) | 145 (35.4) | 507 (28.8) | 474 (12.6) | 129 (13.6) | 200 (21.0) | 422 (10.5) | 12 (7.5) |
| AT | 51 (0.42%) | 1 (0.2) | 3 (0.2) | 17 (0.5) | 7 (0.7) | 4 (0.4) | 19 (0.5) | 0 |
| SVT | 211 (1.76%) | 2 (0.5) | 16 (0.9) | 74 (2.0) | 16 (1.7) | 10 (1.1) | 86 (2.1) | 7 (4.4) |
| MVT | 121 (1.01%) | 1 (0.2) | 8 (0.5) | 49 (1.3) | 16 (1.7) | 4 (0.4) | 41 (1.0) | 2 (1.2) |
| PVT/VF | 25 (0.21%) | 2 (0.5) | 1 (0.1) | 5 (0.1) | 3 (0.3) | 3 (0.3) | 11 (0.3) | 0 |
| 2 Arrhythmias | 2203 (18.33%) | 107 (26.1) | 466 (26.4) | 521 (13.8) | 166 (17.5) | 209 (22.0) | 717 (17.9) | 17 (10.6) |
| 3+ arrhythmias | 1273 (10.6%) | 55 (13.4) | 326 (18.5) | 261 (6.9) | 101 (10.6) | 128 (13.5) | 381 (9.5) | 20 (12.4) |

Abbreviations: AF, atrial fibrillation; Mgmt, management; CS, cryptogenic stroke; PVT, polymorphic ventricular tachycardia; VF, ventricular fibrillation; VT, ventricular tachycardia.

**Supplemental Table 5**. Cardiac ablation procedures inclusive of pre-ICM baseline and post-ICM follow-up periods

|  | **Overall ICM population** | **AF Ablation Monitoring** | **AF Mgmt** | **Cryptogenic Stroke** | **Palpitations** | **Suspected AF** | **Unexplained Syncope** | **VT** |
| --- | --- | --- | --- | --- | --- | --- | --- | --- |
|  | **N=17,037** | **N=431** | **N=2,193** | **N=4,852** | **N=1,252** | **N=1,346** | **N=6,716** | **N=247** |
| **Timing of ablation* procedures, inclusive of pre- and post-ICM periods** |  |  |  |  |  |  |  |  |
| Ablation pre-ICM, n(%) | **1613 (9.5%)** | 173 (40.1%) | 576 (26.3%) | 112 (2.3%) | 123 (9.8%) | 210 (15.6%) | 384 (5.7%) | 35 (14.2%) |
| Ablation concurrent with ICM | **547 (3.21%)** | 206 (47.8%) | 148 (6.7%) | 10 (0.2%) | 28 (2.2%) | 53 (3.9%) | 86 (1.3%) | 16 (6.5%) |
| Ablation post-ICM | **1756 (10.3%)** | 120 (27.8%) | 515 (23.5%) | 192 (4.0%) | 220 (17.6%) | 205 (15.2%) | 472 (7.0%) | 32 (13.0%) |
| **Patients with ≥1 ablation at any time (pre-, during, or post-ICM insertion)** | **3,363 (19.7%)** | **366 (84.9%)** | **1,038 (47.3%)** | **303 (6.2%)** | **333 (26.6%)** | **411 (30.5%)** | **846 (12.6%)** | **66 (26.7%)** |
| **Patients with any re-ablations post-ICM, of those with a history of ablation at any time (above)^1^** | **732 (21.8%)** | 100 (27.3%) | 260 (25.0%) | 41 (14%) | 78 (23%) | 88 (21%) | 150 (18%) | 15 (23%) |
| **Time from ICM implant to re-ablation, months (among patients with ablations during the 1 year prior to or on ICM implant encounter)** |  |  |  |  |  |  |  |  |
| Mean (SD) | **17.1 (14.6)** | 19.5 (15.5) | 18.1 (15) | 14.4 (11.7) | 19.5 (14.9) | 14.1 (12.5) | 10.8 (10.2) | 19.3 (20) |
| Median (IQR) | **13.1 (20.4)** | 6.1 (24.7) | 14 (22.9) | 11 (17) | 17 (23) | 11.3 (15.4) | 8.1 (10.3) | 18.8 (22.3) |
| N | **333** | 86 | 127 | 7 | 26 | 35 | 42 | 10 |
| **Time from 1st ablation to re-ablation, months (among patients with no ablations prior to or on ICM implant date, and an ablation within 1 year post-ICM implant)** |  |  |  |  |  |  |  |  |
| Mean (SD) | **12.5 (14.3)** | 7 (8.3) | 12.4 (14.1) | 12.1 (17.8) | 13.3 (13.4) | 17.6 (14.3) | 9.6 (14.1) | 5 (5.8) |
| Median (IQR) | **7 (22.1)** | 4.3 (1.8) | 6.7 (19.4) | 2 (18.3) | 9 (22.7) | 15.3 (21.1) | 3.3 (11.8) | 3.3 (3.1) |
| N | **195** | 4 | 61 | 20 | 34 | 28 | 44 | 4 |

Abbreviations: AF, atrial fibrillation; ICM, insertable cardiac monitor; IQR, interquartile range; SD, standard deviation; VT, ventricular tachycardia.

*Includes ablations for atrial fibrillation, supraventricular tachycardia, or ventricular tachycardia.

**Supplemental Table 6**. Arrhythmia-related procedure rates in ICM patients with versus without a history of heart failure

| **Procedures post ICM implant** | **Overall ICM population N = 17,037** | **History of HF N = 2,450** | **No history of HF N = 14,587** | **p-value^1^** |
| --- | --- | --- | --- | --- |
| IPG implant, n (%) | **2,404 (14%)** | 491 (20%) | 1,913 (13%) | <0.001 |
| ICD implant, n (%) | **251 (1.5%)** | 99 (4.0%) | 152 (1.0%) | <0.001 |
| CRT implant, n (%) | **202 (1.2%)** | 88 (3.6%) | 114 (0.8%) | <0.001 |
| Cardioversion, n (%) | **921 (5.4%)** | 217 (8.9%) | 704 (4.8%) | <0.001 |
| Ablation*, n (%) | **1,756 (10%)** | 317 (13%) | 1,439 (9.9%) | <0.001 |

Abbreviations: AF, atrial fibrillation; CRT, cardiac resynchronization therapy- with defibrillator (CRT-D) or pacemaker (CRT-P); HF, heart failure; ICD, implantable cardioverter defibrillator; ICM, insertable cardiac monitor IPG, implantable pulse generator.

*Includes ablations for atrial fibrillation, supraventricular tachycardia, or ventricular tachycardia.
